# A patient activation intervention in primary care for patients with chronic pain on long term opioid therapy: results from a randomized control trial

**DOI:** 10.1186/s12913-024-10558-3

**Published:** 2024-01-22

**Authors:** Monique B. Does, Sara R. Adams, Andrea H. Kline-Simon, Catherine Marino, Nancy Charvat-Aguilar, Constance M. Weisner, Andrea L. Rubinstein, Murtuza Ghadiali, Penney Cowan, Kelly C. Young-Wolff, Cynthia I. Campbell

**Affiliations:** 1 Division of Research, Kaiser Permanente Northern California, 2000 Broadway, Oakland, CA 94612-2403 USA; 2 Physical Medicine and Rehabilitation, Kaiser Permanente Northern California, Santa Clara, CA USA; 3 Department of Psychiatry, University of California, San Francisco, CA USA; 4 Department of Pain Medicine, The Permanente Medical Group, Santa Rosa, CA USA; 5 Addiction Medicine and Recovery Services, The Permanente Medical Group, San Francisco, CA USA; 6 American Chronic Pain Association, Rocklin, CA USA

**Keywords:** Chronic pain, Patient activation, Primary care, Pragmatic clinical trial, Prescription opioids

## Abstract

**Background:**

Given significant risks associated with long-term prescription opioid use, there is a need for non-pharmacological interventions for treating chronic pain. Activating patients to manage chronic pain has the potential to improve health outcomes. The ACTIVATE study was designed to evaluate the effectiveness of a 4-session patient activation intervention in primary care for patients on long-term opioid therapy.

**Methods:**

The two-arm, pragmatic, randomized trial was conducted in two primary care clinics in an integrated health system from June 2015—August 2018. Consenting participants were randomized to the intervention (*n* = 189) or usual care (*n* = 187). Participants completed online and interviewer-administered surveys at baseline, 6- and 12- months follow-up. Prescription opioid use was extracted from the EHR. The primary outcome was patient activation assessed by the Patient Activation Measure (PAM). Secondary outcomes included mood, function, overall health, non-pharmacologic pain management strategies, and patient portal use. We conducted a repeated measure analysis and reported between-group differences at 12 months.

**Results:**

At 12 months, the intervention and usual care arms had similar PAM scores. However, compared to usual care at 12 months, the intervention arm demonstrated: less moderate/severe depression (odds ratio [OR] = 0.40, 95%CI 0.18–0.87); higher overall health (OR = 3.14, 95%CI 1.64–6.01); greater use of the patient portal’s health/wellness resources (OR = 2.50, 95%CI 1.42–4.40) and lab/immunization history (OR = 2.70, 95%CI 1.29–5.65); and greater use of meditation (OR = 2.72; 95%CI 1.61–4.58) and exercise/physical therapy (OR = 2.24, 95%CI 1.29–3.88). At 12 months, the intervention arm had a higher physical health measure (mean difference 1.63; 95%CI: 0.27–2.98).

**Conclusion:**

This trial evaluated the effectiveness of a primary care intervention in improving patient activation and patient-reported outcomes among adults with chronic pain on long-term opioid therapy. Despite a lack of improvement in patient activation, a brief intervention in primary care can improve outcomes such as depression, overall health, non-pharmacologic pain management, and engagement with the health system.

**Trial Registration:**

The study was registered on 10/27/14 on ClinicalTrials.gov (NCT02290223).

**Supplementary Information:**

The online version contains supplementary material available at 10.1186/s12913-024-10558-3.

## Introduction

Twenty one percent of adults in the United States had chronic pain (pain > 3 months) in 2021, and chronic pain is a principle reason for primary care visits [1, 2]. Prescription opioids are the most common treatment for chronic pain, with primary care physicians accounting for nearly 45% of all dispensed opioid prescriptions [3]. The prevalence of long-term opioid therapy (LTOT; defined as opioid use more than 90 days [4]) has been estimated at 5.4% in the United States [5]. Although rates of opioid prescribing have declined nationally since 2012 [6], 143 million opioid prescriptions were dispensed in 2020 [6] and 9.2 million people over age 12 misused prescription opioids in 2021 [7]. With questionable long-term effectiveness for chronic pain [8–10] and high risk of harm (e.g., overdose, addiction), physicians and patients are seeking nonpharmacological, behavioral treatment options for chronic pain, but options are lacking [11].

Behavioral strategies to manage chronic pain are not widely available or typically covered by insurance [12, 13]. When available they are usually located in specialty pain management clinics, where stigma and capacity can be barriers to care [14]. Embedding behavioral interventions for chronic pain into primary care may improve acceptability and access [15]. Time commitment can be another barrier, with behavioral interventions for chronic pain often necessitating up to eight 2-h sessions [16–19]. Low-intensity options may be more feasible for patients and still effective [20]. Recent studies of less intensive interventions (4–5 sessions) for chronic pain have shown effectiveness for pain severity, pain interference, pain catastrophizing, and depression [21], and a single session pain relief skills class showed comparable efficacy to several sessions of cognitive behavior therapy for pain catastrophizing, pain intensity and pain interference at 3 months [21, 22].

Patient-centered interventions focused on improving patient activation, defined as having the knowledge, skills, and confidence to manage one's health and health care [23] have been shown to improve health outcomes for chronic diseases such as diabetes and arthritis. Yet, there are few behavioral interventions focusing on improving patient activation in patients with chronic pain. In a survey of patients with chronic pain, higher patient activation levels were associated with improved clinical outcomes such as lower pain intensity, improved mood and better quality sleep [24]. Activation appears to be modifiable and could be an important target for chronic pain behavioral interventions [25].

The ACTIVATE trial compared a brief, 4-session patient activation intervention with usual care among individuals with chronic pain on long-term opioid therapy treated in primary care. The intervention was intended to promote patient activation by helping participants gain the knowledge, skills, and confidence needed to manage chronic pain. The study team hypothesized that compared to usual care participants, intervention arm participants would have higher levels of patient activation (primary outcome measured with the patient activation measure, PAM) [26], more engagement with the health system, improved quality of life, function, and mood, and reduced prescription opioid use (secondary outcomes) at 12 months. To our knowledge no prior studies have evaluated the PAM and other study outcomes in a low-intensity primary care intervention focused exclusively on activating patients with chronic pain on long-term opioids and involving significant stakeholder engagement.

## Methods

### Setting and study design

The study was conducted in two large, diverse primary care clinics in Kaiser Permanente Northern California (KPNC), an integrated health care system with > 4.5 million members [27]. The demographic and socioeconomic characteristics of KPNC members are broadly representative of the insured, regional population [28]. The study was approved by the institutional review board of KPNC and all study participants signed an informed consent form and authorization to use and disclose protected health information prior to participation. The study was registered on ClinicalTrials.gov on 10/27/14 (NCT02290223) and followed the Consolidated Standards of Reporting Trials (CONSORT) reporting guidelines for randomized trials [29]. The study design was a two-arm, pragmatic, randomized trial conducted from June 2015—August 2018. This pragmatic trial was embedded in primary care under usual conditions and accessible to patients with a range of pain severity and pain conditions; exclusions were minimized [30, 31].

### Stakeholder engagement

Using the Patient-Centered Outcomes Research Institute (PCORI) engagement rubric as a guide [32, 33], a diverse panel of patient and clinical stakeholders was regularly convened. Stakeholders participated in all stages of the ACTIVATE study: study design, participant recruitment and retention, development of intervention curriculum, as well as data interpretation and dissemination of findings. Of note, patient stakeholders were involved in identification and selection of patient-centered study outcomes that were relevant and meaningful to patients, such as function, social roles, depression, and sleep.

### ACTIVATE Intervention

The ACTIVATE intervention aimed to ‘activate’ participants to engage in their pain management more fully. It consisted of four 90-min group sessions conducted by a licensed psychologist (CM) with expertise in chronic pain. Groups were intentionally small (3–8 participants) to facilitate discussion and interaction. Each session included an educational presentation and skills practice (e.g., goal setting, guided imagery, patient-provider communication role play, patient portal navigation). The intervention was designed to be brief (4 weekly sessions), easily accessible (embedded in primary care), and portable (transportable to other health systems). The intervention was not adapted to individual participant needs but was designed to support participants with a range of pain types and severity (e.g., patients with less severe pain conditions and those transitioning from acute to chronic pain), and to serve as a steppingstone to specialized treatment for those with higher pain severity. After each session, the study psychologist self-assessed fidelity to monitor consistent delivery of content.

The ACTIVATE intervention was based on work by Bernabeo and Holmboe [34], by Hibbard [23, 25], and previous work of the research team linking patients receiving addiction treatment to primary care [35, 36]. The Bernabeo and Holmboe framework focuses on patient competencies that activate and empower patients to take steps to engage in their health and health care [34]. Patient competencies include developing patient-physician communication skills and accessing and utilizing information and services. Specifically, the curriculum (Table 1) incorporated motivational interviewing and cognitive behavioral strategies to build patient competencies of health literacy, shared decision-making skills, use of the health system’s online patient portal and health education resources, as well as provide education on the neurophysiology of pain and the consequences of long-term prescription opioid use.

**Table 1:** ACTIVATE intervention curriculum

Session #	Session topics
Session 1	Patients’ perceptions of their role in their health care Difficulty talking with providers about opioids and pain Mind–body framework to pain management Education about pain and opioids for treatment of pain
Session 2	Healthy behaviors/lifestyle and how relates to pain health Communicate priorities to physicians with readiness ruler Physiological self-regulation skills such as diaphragmatic breathing and autogenics Stress management skills
Session 3	Practice logging onto patient portal View test results/labs, book appointments, etc Online wellness programs, e.g., mindfulness and sleep Accessing complementary care, e.g., acupuncture, nutrition
Session 4	How to collaborate with physician Priority setting with readiness ruler Assertive communication skills Using “My Care Plan” to communicate with physician Importance of planning for periods of increased stress

### Usual care

Participants randomized to usual care received the standard of care as determined by their primary care providers. Usual care was selected as the appropriate comparator since there is no established model of care for patients in primary care on long-term opioid therapy; providers may refer patients with long-term, severe pain to specialty pain programs or suggest a range of pharmacological and non-pharmacological treatments.

### Study participants, eligibility and recruitment

Adult patients (age ≥ 18) at two primary care clinics with ≥ 3 cumulative days’ supply of opioid medication dispensed each week during the previous 3-month period were identified through the electronic health record (EHR). This criterion was developed in consult with a chronic pain physician (AR) to ensure that we identified patients with consistent opioid use for at least 3 months, which aligns with long-term opioid therapy definitions [4]. The inclusion criteria were intentionally broad to accommodate the diversity of patients with chronic pain seen in primary care and were not based on acuity.

Participants were excluded based on chart review if they 1) had a malignant cancer diagnosis, 2) did not read or speak English, 3) were no longer taking opioids (or planned to taper within 30 days), 4) were in hospice or end of life palliative care, 5) were enrolled in a chronic pain management or addiction treatment program, or 6) had cognitive impairment or a serious physical or psychiatric comorbidity that would preclude participation in the behavioral intervention. Primary care providers reviewed and excluded potentially eligible patients if they were not appropriate for the intervention. Participants who met EHR eligibility criteria and provider approval were sent an invitation letter, and further screened for eligibility with a follow-up phone call. A research assistant conducted informed consent, baseline survey, and randomization at a subsequent in-person appointment at the primary care clinic.

### Randomization

Consenting participants were randomized (1:1) to the ACTIVATE intervention or usual care using a block randomization design with block size of 10. Randomization was performed using a random number generator and allocation was maintained with sealed, opaque envelopes. Allocation was revealed to participants and research staff after enrollment to avoid potential ascertainment bias.

### Data collection

Study participants completed online surveys at baseline (self-administered in-person) and at 6- and 12- months (interviewer-administered by telephone) (See questionnaires, Additional files 1, 2 and 3). In addition to study-developed questions (e.g., health care utilization, chronic pain management), the surveys utilized standardized, validated instruments whenever possible. Data on prescription opioid use was extracted from the EHR. Participants were provided with $50 compensation for each survey ($150 total). After enrollment, participants were not required to be active health system members or to complete all 4 intervention sessions to participate in the follow-up interviews.

### Outcome measures

Patient-centered outcome measures were selected based on previous research and stakeholder priorities.

### Primary outcome

#### Patient activation

Patient activation was measured by the Patient Activation Measure (PAM-13), a 13-item instrument measuring patient beliefs, knowledge, and confidence in their management of health-related tasks [23, 26]. Participants indicated their level of agreement to 13 statements with 1 = strongly disagree and 4 = strongly agree. The item points were summed (range 0–52) and normalized to 100-point scores, with higher scores related to higher activation levels. An increase in 3 to 6 points over time has been associated with improved health behaviors and outcomes [37].

### Secondary outcomes

#### Depression

Depression was measured using the Patient Health Questionnaire-9 (PHQ-9), a reliable and well-validated instrument used to screen and diagnose depression severity [38]. Scores ranged from 0–27 with higher scores indicating more severity. Scores were dichotomized with a score ≥ 10 indicating moderate to severe depression [39].

#### Quality of life

The Patient-Reported Outcomes Measurement Information System (PROMIS) Global Health measure assessed general perceptions of health and quality of life [40]. The 10 items represented two dimensions, Mental Health and Physical Health, and included questions on overall physical health, mental health, social health, pain, fatigue, and overall perceived quality of life. Raw scores for Global Mental and Physical Health were converted to standardized T-scores [41]. Higher scores represent better health and quality of life.

#### Overall health

Overall health status was represented by a single item on the PROMIS Global Health measure (“In general, would you say your health is…?”) [40]. Answers are reported as a raw score with range 1–5, with 1 = poor to 5 = excellent [40].

#### Pain intensity

A single item on the PROMIS Global Health measure assessed pain intensity (“How would you rate your pain, on average?”) [40]. Pain level is reported as a raw score with range 1–10. The published scale has a range of 0–10; however, a 1–10 scale was inadvertently used on the survey, with 1 = no pain to 10 = worst imaginable pain.

#### Prescription opioid use

Prescription opioid use was assessed through pharmacy dispensation data from the EHR (including claims for fills outside of KPNC pharmacies) and converted into morphine milligram equivalent (MME) by multiplying the quantity of each prescription (e.g., days’ supply) by the strength of prescription (milligrams of opioid/unit dispensed). The resulting product was then multiplied by the conversion factor for MMEs [42, 43]. We calculated the average daily MME dispensed for the 6-month period (defined as 183 days) preceding the 12-month interview by summing the MMEs for the prescriptions dispensed and dividing by 183.

#### Functional status

Single items on the PROMIS Global Health measure assessed self-reported performance on social activities and roles (“In general, please rate how well you carry out your usual social activities and roles?”) and performance of everyday physical activities (“To what extent are you able to carry out your everyday physical activities such as walking, climbing stairs, carrying groceries, or moving a chair?”) [40]. Answers were reported as raw scores with range 1–5, and then converted to standardized T-scores [41]. Higher scores indicate better function.

#### Use of online patient portal

Participants reported at baseline if they ever attended a KPNC health education class (yes/no) and if they ever used the online patient portal (yes/no) and if yes, how they used it (e.g., emailed physician, checked lab results or immunization history, used health and wellness resources). At the 6- and 12-month follow-up interviews, participants were asked about portal use in the previous 6 months.

#### Pain management strategies

Participants indicated if they used: opioid medication prescribed by a doctor; non-opioid medication prescribed by a doctor; over the counter medication; complementary/alternative medicine; meditation, relaxation, or mindfulness practice; pain classes or therapy; massage or other bodywork; and exercise, stretching or physical therapy. All measures were dichotomous.

#### Pain coping

The Chronic Pain Coping Inventory (CPCI) assessed positive and negative behavioral and cognitive coping strategies [44]. We used the abbreviated 42-item CPCI, which contains eight subscales: guarding, resting, asking for assistance, relaxation, task persistence, exercising/stretch, coping self-statements, and seeking social support [45]. For each subscale, the participant was asked the number of days (0–7 days) they performed each task. Scores, ranging from 0–7, were calculated separately for each of the eight subscales.

### Statistical analysis

We used an intent-to-treat approach with the full sample and examined baseline characteristics of the intervention and usual care groups. Baseline characteristics were compared using chi-squared tests for categorical measures and t-test for continuous measures. For outcome measures at baseline and 12 months, we report percentages for binary outcomes and mean/standard deviation for continuous and ordinal measures.

Between group differences in the outcome measures at 12 months were analyzed. For observed differences in continuous and ordinal outcome measures, we report the mean differences and p-values of the t-test. For observed differences in the dichotomous measures, we report the difference in the proportion of the outcome variable between the two groups (SAS FREQ RISKDIFF option) and the p-value of the chi-square test. For estimated differences, continuous outcome variables were analyzed using linear mixed-effects models with random intercepts; dichotomous 12-month outcome variables were analyzed with non-linear mixed-effects models with logit link and random intercepts; and ordinal measures (i.e., overall health, social activity/roles, physical activity) were analyzed with non-linear mixed-effects models with cumulative logit link and random intercepts. Each participant had three repeated measures (baseline, 6 months and 12 months). We implemented multiple imputation methods for missing PAM-13 scores at 6 months (*n* = 22 missing) and 12 months (*n* = 34 missing) using SAS procedures MI and MIANALYZE. This technique created 30 complete datasets, all with plausible values for each missing value, which were analyzed using the modeling approach described below. PROC MIANALYZE was then used to combine results from the 30 datasets to generate valid estimates and adjust standard errors for inference.

All models included an indicator variable for treatment arm (1 = intervention; 0 = usual care), time as continuous variable (0 = baseline, 1 = 6 months, 2 = 12 months), and a term for the interaction of treatment by time. We adjusted for two measures (CPCI-42 relaxation and CPCI-42 exercise/stretch) that were statistically different at baseline. We used a repeated measures mixed effects framework as detailed above to examine differences in the 12-month outcomes by treatment arm over time by including an interaction of time and study arm. Repeated measures models were presented for the primary outcome (PAM-13 scores) and secondary outcomes that were significantly different in the bivariate analyses or were of high clinical interest. SAS procedures MIXED, NLMIXED, and GLIMMIX were used.

In addition to the intent-to-treat analysis with the full sample, we conducted per protocol bivariate analyses, which included only participants in the intervention arm who completed all 4 sessions of the intervention (*n* = 120). We conducted the same bivariate analyses on this sample, comparing 12-month outcomes for usual care (*n* = 187) and intervention arm participants.

Our final sample size of 376 eligible patients with an estimated correlation of 0.3 between the 3 repeated measures gave us adequate power (power of 0.938) to detect a small to medium effect size of a 24% difference in standard deviation units in PAM scores (the primary outcome). All tests were two-sided, and statistical significance was defined as *P*< 0.05. We did not adjust for multiple comparisons as we were interested in specific associations between the intervention and outcomes and not the global null hypothesis [46, 47]. However, findings with *P*-values close to 0.05 should be viewed cautiously given the number of secondary outcomes that were included in this study. All outcomes were specified a priori. Analyses were conducted using SAS version 9.3.

## Results

### Participants

**Fig. 1:**
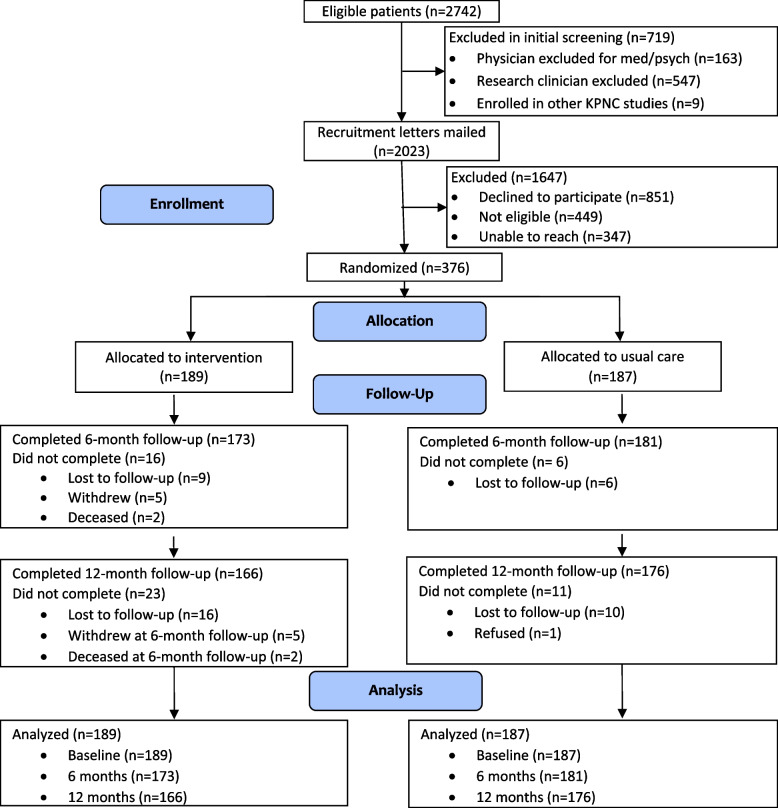
CONSORT diagram of recruitment and retention in the ACTIVATE study. Notes: KPNC = Kaiser Permanente Northern California

A total of 2742 patients met initial eligibility criteria. Of these, 2023 were invited to participate and 376 enrolled. Figure 1 presents the CONSORT diagram and participant flow. Among the 376 enrolled participants, 354 (94%) and 342 (91%) completed the 6-month and 12-month follow-up assessments, respectively. Of the 189 participants in the intervention group, 120 (64%) attended 4 sessions, 23 (12%) attended 3 sessions, 14 (7%) attended 1–2 sessions, and 32 (17%) did not attend any sessions. Patients reported that non-participation was due to lack of transportation, work and childcare responsibilities, and poor health.

Of the 376 enrolled, 58.2% were female, 67.6% were non-Hispanic White, and the mean age was 59.8 years (SD = 13.1) (Table 2). On average, participants experienced pain for 14.6 years (SD = 12.2) and reported taking prescription opioids for an average of 9.0 years (SD = 8.3). Most study participants (87.7%) reported having more than one pain condition and 17.6% reported widespread pain (fibromyalgia). The most common pain types reported were back pain and leg pain (including feet and knee) with 78.7% and 70.7% of participants reporting, respectively. At baseline, mean pain intensity score was 6.7 (SD = 1.5), and the mean daily MME was 34.0 (SD = 57.8) (Table 2). We found no significant differences between study arms in age, sex, race/ethnicity, annual household income, education, marital status, employment status, pain intensity or MMEs (Table 2). However, intervention participants had higher scores on the relaxation (mean 2.4 [SD = 1.9] vs 2.0 [SD = 1.8], *P* = 0.04) and exercise/stretching (mean 3.0 [SD = 2.2] vs 2.6 [SD = 1.9], *P* = 0.04) domains of the CPCI-42 compared to usual care (data not shown). There were no significant differences between the study arms at baseline in all other outcomes.

**Table 2:** Baseline characteristics of study participants, by intervention and usual care

**Characteristic, no. (%)**	**Total (*n* = 376)**	**Intervention (*n* = 189)**	**Usual care (*n* = 187)**	***P***^**a**^
Age, years, mean (SD)	59.8 (13.1)	58.8 (13.7)	60.7 (12.4)	.16
Female sex	219 (58.2)	114 (60.3)	105 (56.1)	.41
Race/ethnicity^b^				.56
Black	20 (5.3)	11 (5.8)	9 (4.8)	
Asian	19 (5.1)	7 (3.7)	12 (6.4)	
Hispanic	63 (16.8)	30 (15.9)	33 (17.6)	
Native American	16 (4.3)	11 (5.8)	5 (2.7)	
Non-Hispanic White	254 (67.6)	128 (67.7)	126 (67.4)	
Annual household income > $50,000	222 (59.0)	112 (59.3)	110 (58.8)	.69
Education				.21
≤ High school graduate or GED	132 (35.2)	58 (30.7)	74 (39.6)	
Associate degree or technical school	115 (30.7)	63 (33.3)	52 (27.8)	
College or higher	128 (34.1)	68 (36.0)	60 (32.1)	
Married	238 (63.3)	116 (61.4)	122 (65.2)	.44
Employed	165 (43.9)	86 (45.5)	79 (42.2)	.52
Average daily opioid dose, MME/day, mean (SD)^c^	34.0 (57.8)	35.8 (68.9)	32.1 (43.8)	.54
Pain				
Pain intensity scale (range 1–10), mean (SD)^d^	6.7 (1.5)	6.7 (1.4)	6.7 (1.6)	.83
Number of pain conditions^e^				.98
1	46 (12.3)	23 (12.2)	23 (12.3)	
≥ 2	329 (87.7)	165 (87.8)	164 (87.7)	
Types of pain conditions				
Back	296 (78.7)	149 (78.8)	147 (78.6)	.96
Legs, feet, and knees	266 (70.7)	133 (70.4)	133 (71.1)	.87
Neck	175 (46.5)	96 (50.8)	79 (42.2)	.10
Widespread pain (or fibromyalgia)	66 (17.6)	34 (18.0)	32 (17.1)	.82
Other	302 (80.3)	152 (80.4)	150 (80.2)	.96

*Abbreviations*: *SD* standard deviation, *MME* morphine milligram equivalents. *GED* General Educational Development exam

^a^Chi-square or t-test

^b^Other categories not reported due to small cell size

^c^Average daily opioid dose based on the 6 months prior to baseline ascertained from pharmacy records

^d^We inadvertently used a 1–10 scale when the published scale is 0–10. We report the mean and standard deviation, and the altered scale was used by both treatment arms

^e^One person was excluded because they marked “Refused/Don’t know” for pain conditions

### Primary outcome measure

Mean Patient Activation Measure (PAM-13) scores were similar between the two study arms at baseline (65.8 vs 65.2) and 12 months (67.7 vs 66.6) (Table 3). The repeated measures mixed effects model (Table 3) found no effect of the intervention on patient activation scores at 12 months (mean difference = -0.10, 95% CI = -2.97- 2.78).

**Table 3:** Outcomes at baseline and 12 months and between-group differences at 12 months

Outcome measure modeled as:	Inter-vention^a^	Usual care^b^	Between-group differences for intervention vs usual care at 12 months			
			**Observed**		**Estimated**^**c**^	
**Continuous outcomes**	**Mean (SD)**	**Mean (SD)**	**Differences in Means (95% CI)**	**P **^**d**^	**Differences in Means (95% CI)**	**P**
**PAM-13 score**						
Baseline	65.8 (15.0)	65.2 (15.9)				
12 months	67.7 (14.8)	66.6 (14.0)	1.07 (-2.01 to 4.14)	.49	-0.10 (-2.97 to 2.78)	.95
***PROMIS—Quality of Life***^***e***^						
**Global Physical Health**						
Baseline	38.3 (6.3)	38.2 (6.1)				
12 months	40.9 (7.6)	39.0 (6.3)	1.97 (0.49 to 3.45)	.01	1.63 (0.27 to 2.98)	.02
**Global Mental Health**						
Baseline	45.8 (9.4)	45.1 (8.3)				
12 months	47.8 (8.0)	46.9 (8.3)	1.61 (-0.08 to 3.30)	.06	1.33 (-0.37 to 3.03)	.12
**Pain intensity (scale 1–10)**						
Baseline	6.7 (1.4)	6.7 (1.6)				
12 months	5.8 (2.1)	6.1 (1.7)	-0.28 (-0.69 to 0.12)	.22	-0.28 (-0.63 to 0.07)	.12
**Prescription opioid use, MME/day**						
Baseline	35.8 (68.9)	32.1 (43.8)				
12 months	28.0 (70.7)	25.3 (32.9)	2.73 (-8.79 to 14.23)	.64	4.08 (-6.97 to 15.13)	.47
**Binary outcomes**	**N (%)**	**N (%)**	**Differences in Proportion (95% CI)**	**P **^**f**^	**Odds Ratio (95% CI)**	**P**
**Moderate to severe depression**^**g**^						
Baseline	49 (25.9)	46 (24.7)				
12 months	25 (15.1)	45 (25.6)	-0.11 (-0.19 to -0.02)	.02	0.40 (0.18 to 0.87)	.02
***Use of Patient Portal***						
**Used health and wellness resources**						
Baseline	47 (25.0)	42 (22.6)				
12 months	76 (45.8)	50 (28.4)	0.17 (0.07 to 0.27)	< .001	2.50 (1.42 to 4.40)	.002
**Checked labs or immunization history**^h^						
Baseline	160 (85.1)	157 (84.4)				
12 months	133 (80.1)	118 (67.0)	0.13 (0.04 to 0.22)	.006	2.70 (1.29 to 5.65)^**i**^	.008^**i**^
***Pain Management Strategies***						
**Meditation, relaxation, or mindfulness**						
Baseline	64 (33.9)	50 (26.7)				
12 months	61 (36.7)	34 (19.3)	0.17 (0.08 to 0.27)	< .001	2.72 (1.61 to 4.58)	< .001
**Exercise, stretching or physical therapy**						
Baseline	108 (57.1)	95 (50.8)				
12 months	127 (76.5)	100 (56.8)	0.20 (0.10 to 0.29)	< .001	2.24 (1.29 to 3.88)	.004
**Ordinal outcomes (scale 1–5)**	**Mean (SD)**	**Mean (SD)**	**Differences in Means (95% CI)**	**P **^**d**^	**Odds Ratio (95% CI)**	**P**
**Overall health**						
Baseline	2.7 (0.9)	2.6 (0.9)				
12 months	2.7 (0.9)	2.4 (0.9)	0.29 (0.09 to 0.49)	.004	3.14 (1.64 to 6.01)	< .001
***Function***						
**Social activity and roles**						
Baseline	3.3 (1.1)	3.1 (1.1)				
12 months	3.3 (1.0)	3.1 (0.9)	0.24 (0.04 to 0.44)	.02	1.51 (0.91 to 2.51)	.11
**Everyday physical activities**						
Baseline	3.4 (1.0)	3.3 (1.0)				
12 months	3.6 (1.1)	3.3 (1.1)	0.28 (0.04 to 0.51)	.02	1.54 (0.89 to 2.68)	.13

*Abbreviations*: *SD* standard deviation. *CI* confidence interval. *PAM-13* Patient Activation Measure-13, PROMIS *Patient-Reported Outcomes Measurement Information System,* MME morphine milligram equivalents

^a^*N* = 189 at Baseline; *N* = 166 at 12 months

^b^*N* = 187 at Baseline; *N* = 176 at 12 months

^c^Modeled with generalized estimating equation (GEE); Outcomes were modeled separately; The independent variables in the models of study outcomes were time, intervention, the interaction of time intervention, baseline Chronic Pain Coping Inventory-42 (CPCI-42) relaxation, and baseline CPCI-42 exercise (CPCI relaxation and exercise were statistically different in the study arms at baseline)

^d^T-test

^e^PROMIS Global Health raw scores are converted to T-scores, with a mean of 50 and a standard deviation of 10

^f^Chi-square

^g^Moderate to severe depression as measured by a Patient Health Questionnaire-9 score ≥ 10

^h^For this self-reported measure at baseline, patients were asked if they ever used the service. At the 12-month follow-up survey, patients were asked about their use in the previous 6 months. Since the ‘ever used’ proportion was higher than the 12-month proportion, baseline values were excluded from the model and only the 6th and 12th month values were used

### Secondary outcomes

The intervention group demonstrated better secondary outcomes at 12 months compared to the usual care group (Table 3): lower odds of moderate to severe depression (OR = 0.40, 95%CI 0.18–0.87); higher self-reported overall health (OR = 3.14, 95%CI 1.64–6.01); greater use of the patient portal to access health/wellness resources (OR = 2.50, 95%CI 1.42–4.40) and check lab results/immunization history (OR = 2.70, 95%CI 1.29–5.65); and greater use of meditation (OR = 2.72; 95%CI 1.61–4.58) and accessing exercise/physical therapy resources (OR = 2.24, 95%CI 1.29–3.88). At 12 months, the intervention group also had a higher PROMIS Global Physical Health T-Score (mean difference 1.63; 95%CI: 0.27–2.98).

### Per protocol analyses

The bivariate per protocol results were not different from the intent-to-treat results with the exception of the pain intensity outcome. In the intent-to-treat analysis, there was not a significant difference in pain intensity at 12 months (*P* = 0.22, Table 3). However, in the per protocol analysis, pain intensity was significantly lower at 12 months for the intervention arm (5.6 ± 2.1) compared to the usual care arm (6.1 ± 1.7, *P* = 0.03 data, not shown).

## Discussion

This patient-centered study examined the effectiveness of a brief, primary care-based patient activation intervention for patients on LTOT. Results did not support our primary hypothesis that the ACTIVATE intervention would result in higher patient activation. There was no significant difference in pain intensity between study arms, however there was a benefit over time for patient-centered outcomes such as depression and physical/overall health. Intervention participants used the patient portal more, as well as self-care strategies such as mindfulness, meditation and relaxation, and exercising and stretching.

Findings for our primary outcome are consistent with a 2018 study by Nøst et al. which did not find a long-term effect on patient activation in a self-management intervention with people with chronic pain. [48] Although some individual studies have shown positive PAM-13 results [49–51], a recent meta-analysis of randomized trials of interventions for chronic conditions did not show a change in patient activation [52]. In people with chronic health conditions, higher activation levels have been associated with more direct support from health care providers [53]; therefore patient activation levels in this study may have been higher if the intervention had been led or initiated by a primary care doctor or a member of a patient’s care team, rather than a research health educator. Due to the complex nature of chronic pain, sustained activation might be best achieved through an interdisciplinary team approach where behaviors are supported and reinforced by multiple care providers in a variety of settings [54].

Although we hypothesized that a brief (4-session) intervention would lead to increased activation in patients with chronic pain, it is possible that 4 sessions were not enough to impact activation. The curriculum was designed to support patients across a range of pain types and severity, with the theory that more severe patients may be activated to pursue further appropriate treatment. However, patient heterogeneity may have limited the relevance of the curriculum to some participants and may have diminished the potential to observe activation. Improving and sustaining PAM scores may necessitate a more adaptive, as well as extended, intervention that can be individualized to patient needs. In addition, moderate adherence to the intervention (64% attended all 4 sessions) could have led to smaller than expected changes in activation level; however, results from per protocol analyses were consistent, making this concern less likely.

Previous studies have found that interventions may be more effective for patients with low baseline activation levels [55]. Study results may have been impacted by patient’s underlying level of activation, which was slightly higher (65.5) relative to general United States population (61.6) [23]. The PAM-13 can be a useful tool for tailoring interventions [56–58]. If the ACTIVATE intervention had been tailored to patient’s baseline activation level (e.g., longer/more frequent sessions for patients with lower baseline activation), it may have led to more substantial differences in activation over time. Further research is needed to examine how patient activation can be used to develop personalized treatment plans and interventions for patients with chronic pain.

Patient activation can also be more challenging among older adults, despite the potential for health improvements [59, 60]. The study’s average age of 60 years and an average pain duration of 15 years might have presented greater challenges to impacting patient activation than in other chronic disease populations. It is also important to note that patients with chronic pain suffer from additional stigma relative to patients with other chronic diseases– the risks of misuse and addiction associated with prescription opioid use are often portrayed in the media. Patients with chronic pain have often felt dismissed and their concerns minimized by the medical system, and societal pressures to reduce prescription opioid use have increased. Confidence is a key construct underlying the PAM, yet chronic pain patients may have a harder time feeling increased confidence in managing their health, given negative experiences in the health system, and the lack of control they have experienced around managing their pain.

Although our primary hypothesis was not supported, several other important findings emerged. There were notable differences over time between the two arms in depression. Participants in the intervention arm reported lower likelihood of moderate to severe depression scores at 12 months and a steeper decrease from baseline to 12 months in the prevalence of moderate to severe depression compared to the usual care group. While the intervention did not focus on depression per se, elements of the curriculum (e.g., support from peers in the group, access to resources such as the intervention clinician) could have impacted depression symptoms. Depression is highly comorbid with prescription opioid use [61] and chronic pain [62] and chronic pain is associated with poorer depression outcomes. Chronic pain interventions that impact depression are critical to improving patient-centered outcomes for people with chronic pain.

Participants in the intervention arm also more frequently employed exercise, stretching, or physical therapy as a pain management strategy, and reported improved physical health and overall health over time. Our results also demonstrated that intervention participants had increased use of self-care strategies such as meditation, relaxation, or mindfulness. A large part of the intervention was dedicated to promoting healthy behaviors, and correlating lifestyle choices with pain levels. Education around the mind–body connection and the neurophysiology of pain and the curriculum’s focus on promoting sleep, exercise, and nutrition may have contributed to improved self-care and physical and overall health even if not achieved through increased activation. There was no significant difference in pain intensity over time between the two study arms. However, pain intensity alone does not capture the complexity of the pain experience. Functional measures of physical and social health are critical to assess in people with chronic pain [63], and are reliable indicators for monitoring overall quality of life.

Participants in the intervention arm reported increased use of the patient portal for accessing health and wellness resources, checking lab results, and immunization history, consistent with our hypothesis of greater engagement with the health system. Online tools and patient portals provide unique opportunities to engage patients in their health care, by facilitating communication with providers and improving access to online wellness resources, which may be particularly useful for patients with limited mobility. Overall, elements of the ACTIVATE study’s curriculum that were more experiential and interactive (e.g., mindfulness, accessing online patient portal) may have been more sustainable than strategies that were discussed but not modeled or practiced in the sessions [64]. Additionally, because primary care physicians treat the majority of chronic pain patients, embedding interventions in primary care has the potential to improve their effectiveness by increasing access.

Both study arms experienced a decrease in opioid use over the study period, and there was no significant benefit for participants in the intervention. This decrease likely reflects the changing prescribing environment nationally and within the health system, which had implemented a safe opioid prescribing initiative to reduce higher risk prescribing practices during the study timeframe [65, 66]. The impact of these policies likely subsumed any potential benefit of the intervention in the absence of these initiatives.

The study had limitations. It was conducted in an integrated health care system in Northern California, which limits generalizability. However, members are representative of the insured population, and the clinical characteristics of individuals with chronic pain are typically similar in different health care settings. The intervention curriculum was designed to be replicable and scalable to a wide range of health care organizations. Participant adherence to the protocol was challenging with 75% of intervention participants completing 3 or more sessions. However, there were no marked differences in the bivariate results between the intent-to-treat analyses and per protocol analyses. Opioids obtained outside of the health system that KPNC did not pay for were not captured, although given financial advantages most members fill medications in the health system [67, 68].

The study had several strengths. To our knowledge, this is the only study that evaluates the effectiveness of a brief, patient activation intervention in primary care patients with chronic pain on long-term opioids. It includes a large sample with adequate statistical power to detect clinically meaningful effects, a pragmatic study design embedded in primary care with minimal exclusions, inclusion of EHR data, long-term follow-up, and low rates of discontinuation (91% completed the 12-month follow up assessment).

## Conclusion

Patients with chronic pain need effective, patient-centered strategies to reduce the significant risks associated with long-term prescription opioid use, particularly during a time when prescription opioids are more restricted. Behavioral interventions embedded in primary care can improve access and address the strong desire of patients and primary care physicians for support. This trial demonstrated that a brief primary care activation intervention can improve and, in some cases, sustain patient-centered outcomes such as depression and overall and physical health that are critical indicators of quality of life and important to both patients and clinicians, even without a measurable increase in activation. Further, our positive findings for self-care strategies and patient portal use suggest that patients are open to non-pharmacological alternatives to managing their pain.

### Supplementary Information

**Additional file 1.** ACTIVATE study baseline questionnaire. Self-administered online survey completed at baseline/enrollment.

**Additional file 2. **ACTIVATE study 6-month questionnaire. Interview-administered survey completed over telephone at 6-months post-enrollment.

**Additional file 3.** ACTIVATE study 12-month questionnaire. Interview-administered survey completed over telephone at 12-months post-enrollment.

## Data Availability

De-identified datasets are available upon reasonable request from the corresponding author for the purposes of replicating procedures and results.

## Abbreviations

CI: Confidence interval
CONSORT: Consolidated Standards of Reporting Trials
CPCI: Chronic Pain Coping Inventory
EHR: Electronic health record
GED: General Educational Development exam
GEE: Generalized estimating equation
KPNC: Kaiser Permanente Northern California
LTOT: Long-term opioid therapy
MME: Morphine milligram equivalent
OR: Odds ratio
PAM-13: Patient Activation Measure
PCORI: Patient-Centered Outcomes Research Institute
PHQ-9: Patient Health Questionnaire
PROMIS: Patient-Reported Outcomes Measurement Information System
SD: Standard Deviation
