# Supplementary material for: A patient activation intervention in primary care for patients with chronic pain on long term opioid therapy: results from a randomized control trial
Source: BMC Health Serv Res. 2024 Jan 22;24:112. doi: 10.1186/s12913-024-10558-3 (PMC10802020; doi:10.1186/s12913-024-10558-3)
Supplement: Supplementary file 1 — Additional file 1. ACTIVATE study baseline questionnaire. Self-administered online survey completed at baseline/enrollment. [file 12913_2024_10558_MOESM1_ESM.pdf]

ACTIVATE: Patients Managing Pain
Baseline Questionnaire (Self-Administered)

ENTERED BY RESEARCH STAFF BEFORE SELF ADMINISTERED SURVEY BEGINS:

PPN: ___ ___ ___ ___ ___

Thank you again for agreeing to be part of the ACTIVATE Study. The following questions will help us describe the group of people who are participating in this study, as a group, rather than you as an individual. Please answer questions as honestly as you can. Your answers are confidential. You can refuse to answer any questions or stop the questionnaire at any time. We will be asking a variety of questions with various different time periods. Please refer to the time period specific to each question. Some of the questions may seem similar; please be assured every question is important.

DEMOGRAPHICS

Please check appropriate answers

1. **D0GENDER -** Gender
1☐ Male
2☐ Female
3☐ Other (e.g., Transgender, Intersex)
Specify: ___________________

2. **D0MASTAT** – Marital status
What is your current marital status?
1☐ Married
2☐ Living with Intimate Partner
3☐ Widowed
4☐ Divorced or separated
5☐ Single, never married

3. **D0ETHNIC** - Ethnicity
Are you of Hispanic or Latino descent?
1☐ Yes
2☐ No
3☐ Not sure

4. **D0RACE -** Race
What racial group(s) best describes your background? (If "yes" to D0ETHNIC, What racial group(s) best describes your background **IN ADDITION TO** Hispanic or Latino?
*Choose **ALL** that apply*
1☐ African-American/Black
2☐ American Indian/Alaskan Native
3☐ Asian/Filipino
4☐ Pacific Islander/Native Hawaiian
5☐ Caucasian/White
6☐ Other (Specify): ___________________
7☐ Not known

5. **D0AGE** - Age
___ ___

6. **D0EMSTAT** - Employment Status
What best describes your current employment status?
1☐ Employed full-time
2☐ Employed part-time
3☐ Unemployed
4☐ Retired
5☐ Fulltime Homemaker **→SKIP TO D0EDUC**
6☐ Student (Not employed) **→ SKIP TO D0EDUC**
7☐ Disabled

7. **D0EDUC** – Education
Check the box of highest level completed
1☐ 12 years or less: ___ ___
2☐ High School diploma or GED
3☐ A.A., A.S., other vocational program
4☐ B.A., B.S.
5☐ M.A., M.S.
6☐ Ph.D., M.D., J.D.

8. **D0LIVSIT** – Current living situation
What best describes your current living situation?
Check the box that best applies
1☐ House or apartment (you rent or own)
2☐ House or apartment of friend or relative
3☐ Halfway house or residence in therapeutic community
4☐ Single room occupancy (SRO), hotel or motel
5☐ Homeless (streets, shelter, car, no stable arrangement)
6☐ Institution (jail, hospital)
7☐ Other (Specify): ___________________

9. **D0LIVSUP** – Current living support
Who do you currently live with?
1☐ Alone
2☐ With friend(s) or relative(s)
3☐ With partner or spouse
7☐ Other (Specify): ___________________

10. **D0ARMFOR**
Did you/do you currently serve in the Armed Forces for the United States?
1☐ Yes
2☐ No **→ SKIP TO D0INCOME**

11. **D0INCOME**
Yearly household income after taxes
Check the box that applies

1☐	Less than $10,000	7☐	$60,001 - $70,000
2☐	$10,001 - $20,000	8☐	$70,001 - $80,000
3☐	$20,001 - $30,000	9☐	$80,001 - $90,000
4☐	$30,001 - $40,000	10☐	$90,001 - $100,000
5☐	$40,001 - $50,000	11☐	over $100,000
6☐	$50,001 - $60,000	97 ☐	Rather not say

PATIENT ACTIVATION (PAM)

Now we will ask you some questions about how you feel about your involvement in your own healthcare.

12. Below are some statements that people sometimes make when they talk about their health. Please indicate how much you agree or disagree with each statement as it applies to you personally by checking your answer.

Question	Disagree Strongly	Disagree	Agree	Strongly Agree
**12A. P0HLTHRESP** When all is said and done, I am the person who is responsible for taking care of my health	1☐	2☐	3☐	4☐
**12B. P0HTLTH ACT** Taking an active role in my own health care is the most important thing that affects my health	1☐	2☐	3☐	4☐
**12C. P0HLTH CON** I am confident I can help prevent or reduce problems associated with my health	1☐	2☐	3☐	4☐
**12D. P0KNOWRX** I know what each of my prescribed medications do	1☐	2☐	3☐	4☐
**12E. P0GODOC** I am confident that I can tell whether I need to go to the doctor or whether I can take care of a health problem myself	1☐	2☐	3☐	4☐
**12F. P0TELLDOC** I am confident that I can tell a doctor concerns I have even when he or she does not ask	1☐	2☐	3☐	4☐
**12G. P0CONFTX** I am confident that I can follow through on medical treatments I may need to do at home	1☐	2☐	3☐	4☐
**12H. P0UNDER** I understand my health problems and what causes them	1☐	2☐	3☐	4☐
**12I. P0TXAVAIL** I know what treatments are available for my health problems	1☐	2☐	3☐	4☐
**12J. P0MAIN** I have been able to maintain (keep up with) lifestyle changes, like eating right or exercising	1☐	2☐	3☐	4☐
**12K. P0PREV** I know how to prevent problems with my health	1☐	2☐	3☐	4☐
**12L. P0SOLU** I am confident I can figure out solutions when new problems arise with my health	1☐	2☐	3☐	4☐
**12M. P0LIFE** I am confident that I can maintain lifestyle changes, like eating right and exercising, even during times of stress	1☐	2☐	3☐	4☐

SATISFACTION WITH CARE

Now we will ask you some questions about your satisfaction with your health care.

13. T0STSOVCARE

How would you rate your satisfaction with your overall health care on a scale of "1" to "10," where "1" is the worst possible care and "10" is the best possible care?

1 2 3 4 5 6 7 8 9 10

|-----------|----------|-----------|-----------|-----------|----------|-----------|----------|-----------|

Worst Possible Care Best Possible Care

Answer: ______

14. T0STSPCP

How would you rate your satisfaction with your primary care physician on a scale of "1" to "10," where "1" is the worst possible care and "10" is the best possible care?

1 2 3 4 5 6 7 8 9 10

|-----------|----------|-----------|-----------|-----------|----------|-----------|----------|-----------|

Worst Possible Care Best Possible Care

Answer: ______

15. T0STSPAINMG

How would you rate your satisfaction with your pain management care on a scale of "1" to "10," where "1" is the worst possible care and "10" is the best possible care?

1 2 3 4 5 6 7 8 9 10

|-----------|----------|-----------|-----------|-----------|----------|-----------|----------|-----------|

Worst Possible Care Best Possible Care

Answer: ______

HEALTH CARE UTILIZATION

The next series of questions ask about your health care use. Please read each statement and respond to the questions to the best of your ability.

16. H0KPCOUNS

In the ***past 12 months,*** have you received advice or counseling from a Kaiser doctor, nurse, health educator, or other Kaiser health care professional about: *Check all that apply*

1☐ Pain management
2☐ Your diet (what you eat)
3☐ Losing weight
4☐ Getting more exercise
5☐ Quitting smoking
6☐ Stress or emotional problems (like depression)
7☐ Health screening tests recommended for you
8☐ No advice or counseling

17. H0COMP

Do you have access to a personal computer?

1☐ Yes, at home
2☐ Yes, at other location
3☐ No

18. H0INTER

Do you have access to the Internet?

1☐ Yes, at home
2☐ Yes, at other location
3☐ No

19. H0KPORG

Have you ever accessed KP.org?

1☐ Yes
2☐ No →**SKIP TO H0HLTHEDKP**
97☐ Don't Know/Refused

20. H0WAYSKP

Please check the boxes of those ways you have used KP.ORG**.**

Check all that apply

1☐ Emailed my doctor or received an email from my doctor
2☐ Checked my lab results or immunization history
3☐ Scheduled or cancelled a medical appointment
4☐ Ordered a prescription refill
5☐ Reviewed information from a past visit
6☐ Used KP.org's Healthy Lifestyle Programs (audio podcasts, recipe blogs, tools/calculators, videos).

21. H0HLTHEDKP

Have you ever attended a Kaiser Health Education Class?

1☐ Yes
2☐ No
97☐ Don't Know/Refused

22. H0KPPCP

Do you have a Kaiser Permanente health provider you consider to be your regular or personal doctor/nurse practitioner?

1☐ Yes
2☐ No
97☐ Don't Know/Refused

23. H0KPHPPM

Do you have a regular Kaiser Permanente health provider for pain management?

1☐ Yes
2☐ No
97☐ Don't Know/Refused

24. **H0ERD1**
During the past year, how many days have you been a patient in a non-Kaiser emergency room that Kaiser did not pay for?
___ ___ ___ Days

CHRONIC PAIN

25. **C0PAIN6MYN**
You are participating in this study because you reported experiencing pain. How long have you been in pain?

_______ months C0PAIN6MYN MON
________years COPAIN6MYN YR

26. **C0CNTPAIN**
Has the pain been intermittent or continuous during this time?
1☐ Intermittent
2☐ Continuous

27. **C0SEEKPTX**
How long have you been seeking treatment for pain?

_______ months C0SEEKPTX MON
________years COSEEKPTX YR

28. **C0OPIRXLNG**
How long have you been taking prescription opioids?

_______ months **C0OPIRXLNG MON**
________years **C0OPIRXLNG YR**

29. **C0CNTOPIRX**
Have you been taking prescription opioids intermittently or continuous during this time?
1☐ Intermittent
2☐ Continuous

30. **C0THKPAINTYPE**
Do you consider yourself a person with acute pain, chronic pain, or both?
1☐ Acute
2☐ Chronic
3☐ Acute and chronic
7☐ Refused/DK

We are interested in your expectations for relief of your pain. Please indicate how much you agree or disagree with each statement.

Question	Disagree Strongly	Disagree	Agree	Strongly Agree
**31. C0TXPAINFR**<br>I expect that treatment will make me pain free.	1☐	2☐	3☐	4☐

**32. C0ACCPPNTX**<br>I accept that I will have some pain with even the best treatment.	1☐	2☐	3☐	4☐

33. C0PRMDXPAIN

What is the primary diagnosis that is causing your pain?

____

____

34. C0PAINTYPE

Please describe the parts of your body where you currently are experiencing pain:

Please check all that apply

1☐ Back pain **C0PAINTYPE_BACK**
2☐ Neck pain **C0PAINTYPE_NECK**
3☐ Headache or migraine **C0PAINTYPE_HEAD**
4☐ Stomach ache or abdominal pain **C0PAINTYPE_STOMACH**
5☐ Pelvic pain, groin pain or painful prostatitis **C0PAINTYPE_PELVIC**
6☐ Pain in your shoulders **C0PAINTYPE_SHOULDER**
7☐ Pain in your hands or arms **C0PAINTYPE_HAND**
8☐ Pain in your hips **C0PAINTYPE_HIPS**
9☐ Pain in your legs, including your feet or knees **C0PAINTYPE_LEGS**
10☐ Chest pain **C0PAINTYPE_CHEST**
11☐ Facial ache or pain, including TMD or TMJ pain **C0PAINTYPE_FACE**
12☐ Widespread pain (or fibromyalgia) **C0PAINTYPE_WIDE**
13☐ Other (Specify): ____________________ **C0PAINTYPE_OTH**
14☐ Worst pain is multiple or combination of sites **C0PAINTYPE_COMB**
97☐ Refused/Don't know **C0PAINTYPE_DK**

35. ASK ONLY IF YES TO C0PAINTYPE_COMB

C0WSTPAIN

If you have pain in more than one location, please indicate the site of your worst pain.

Specify: ____________________

36. C0MDREF

How do you currently manage your pain? *Check all that apply*

1☐ Opioid medication prescribed by a doctor (Some examples of opiate pain medicines are: Codeine, Darvocet, Darvon, Fentanyl, Hydrocodone, Methadone, Morphine, Norco, Oxycodone, Oxycontin, Percocet, Percodan, Propoxyphene, and Vicodin)
2☐ Non-opioid medication prescribed by a doctor
3☐ Over the counter medication (like Tylenol)
2☐ Complementary/Alternative Medicine (i.e., acupuncture, herbs) Specify:____
3☐ Meditation, relaxation, or mindfulness practice
4☐ Pain classes or therapy (group or individual)
5☐ Massage or other bodywork
6☐ Exercise, stretching or physical therapy
7☐ Nothing
8☐ Other Specify:____
97☐ Refused/Don't know

The next questions are about prescription opiate pain medicines that your doctor may have prescribed.

C0PAINMED
37. What prescription opioid pain medications have you taken in the past 3 months?
Check all that apply USE DROP DOWN LIST

C0PMEDDAYS
38. In the past 2 weeks, about how many days did you take prescription opioid pain medicines?
_____ Days
97☐ Refused/Don't know

C0TMPMED
39. In the past 2 weeks, about how many times per day did you usually take prescription opioid pain medicine on the days you used these medicines?
__ __ Times per day
97☐ Refused/Don't know

C0PMEDRX
40. Do you always take your prescription opioid pain medication as prescribed?
1☐ Yes
2☐ No

C0NOPMEDRSN
41. Please describe why you sometimes don't take your prescription opioid medication as prescribed. Please check all that apply:
1☐ Side Effects
2☐ Poor pain control (take more than prescribed because amount prescribed doesn't help)
3☐ Do not need it
4☐ Concern about overuse
5☐ Pain is bad and need more than prescribed
7☐ Other (Specify): __________________

C0PMADSE
42. Considering all of the possible side effects of opiate medicines you may have experienced in the past month, (such as nausea, constipation, grogginess), how bothersome were these side effects?
1☐ Not at all bothersome
2☐ A little bothersome
3☐ Moderately bothersome
4☐ Very bothersome
5☐ Extremely bothersome
97☐ Refused/Don't know

Which two side effects bother you the most?
42A **C0SIDEFMOST1**
Feeling groggy or sluggish
Feeling unstable or dizzy
Feeling confused or disoriented
Constipation
Nausea/vomiting
Nightmares
Itchiness

Difficulty urinating or difficulty with urinary retention
Less interest in sex or other sexual difficulties
Other: specify____________

42B **C0SIDEFMOST2**
Feeling groggy or sluggish
Feeling unstable or dizzy
Feeling confused or disoriented
Constipation
Nausea/vomiting
Nightmares
Itchiness
Difficulty urinating or difficulty with urinary retention
Less interest in sex or other sexual difficulties
Other: specify____________

C0HELPOP
43. Over the past month, how helpful have you found opiate pain medicines in relieving your pain.
1☐ Not at all helpful
2☐ A little helpful
3☐ Moderately helpful
4☐ Very helpful
5☐ Extremely helpful
97☐ Refused/Don't know

C0IMPCUTMED
44. How important is it for you to decrease your opioid use?
1☐ Not at all important
2☐ A little important
3☐ Moderately important
4☐ Very important
5☐ Extremely important
97☐ Refused/Don't know

C0CONFCUTMED
45. How confident are you that you can decrease your opioid use?
1☐ Not at all confident
2☐ A little confident
3☐ Moderately confident
4☐ Very confident
5☐ Extremely confident
97☐ Refused/Don't know

46. **C0LTMGOAL**
What is your long-term goal for using prescription opioids for pain management?
1☐ Stay the same
2☐ Increase use
3☐ Decrease use
4☐ Stop use

C0IMPGOAL

47. How important is it for you to reach your goal?

1☐ Not at all important
2☐ A little important
3☐ Moderately important
4☐ Very important
5☐ Extremely important
97☐ Refused/Don't know

48. C0DOCSTOP

Has your doctor talked to you about stopping or tapering your opioids in the last 12 months?

1☐ Yes
2☐ No

49. C0CUTOPIUSE

Are you currently in the process of tapering (gradually decreasing) your opioid use?

1☐ Yes
2☐ No → skip to **Q51**

50. C0DECPVDOPICUT

Do you feel like a partner with your doctor in this decision to taper your opioid use?

1☐ Yes
2☐ No

51. C0GETOPINKP

Have you tried to obtain opioids from other sources in the last 12 months as result of your KP doctor wanting to decrease your opioid use?

1☐ Yes
2☐ No

52. C0CHGDOCOPICUT

Have you switched doctors in the last months, as result of your KP doctor wanting to decrease your opioid use)?

1☐ Yes
2☐ No

53. C0LFKPOPICUT

Have you thought about leaving Kaiser, as result of your KP doctor wanting to decrease your opioid use?

1☐ Yes
2☐ No

54. C0RXS

During the <u>last 12 months,</u> how many of your own opiate prescriptions did you get filled at **NON-Kaiser** pharmacies (including through **NON-Kaiser** web sites).

___ ___ ___ Prescriptions

SCREENER AND OPIOID ASSESSMENT FOR PATIENTS WITH PAIN (SOAPP-5)

55. Now a few general questions about your mood and drug use.

Question	Never	Seldom	Sometimes	Often	Very Often
A. **S0MOODSWG**<br>How often do you have mood swings?	1☐	2☐	3☐	4☐	5☐
B. **S0SMKWP**<br>How often do you smoke a cigarette within an hour after you wake up?	1☐	2☐	3☐	4☐	5☐
C. **S0MEDNFRX**<br>How often have you taken medication other than the way that it was prescribed?	1☐	2☐	3☐	4☐	5☐
D. **S0USEDRG**<br>How often have you used illegal drugs (for example, marijuana, cocaine, etc.) in the past five years?	1☐	2☐	3☐	4☐	5☐
E. **S0LEGPRB**<br>How often, in your lifetime, have you had legal problems or been arrested?	1☐	2☐	3☐	4☐	5☐

CURRENT OPIOID MISUSE (COMM)

56. Please answer each question as honestly as possible. Keep in mind that we are only asking about the **PAST 30 DAYS**. There are no right or wrong answers. If you are unsure about how to answer the question, please give the best answer you can.

Question	Never	Seldom	Sometimes	Often	Very Often
**A. C0TRBMEMB**<br>In the past 30 days, how often have you had trouble with thinking clearly or had memory problems?	1☐	2☐	3☐	4☐	5☐
**B. C0UNDNTASK**<br>In the past 30 days, how often do people complain that you are not completing necessary tasks? (i.e., doing things that need to be done, such as going to class, work or appointments)	1☐	2☐	3☐	4☐	5☐
**C. C0GTNRXMED**<br>In the past 30 days, how often have you had to go to someone other than your prescribing physician to get sufficient pain relief from medications? (i.e., another doctor, the Emergency Room, friends, street sources)	1☐	2☐	3☐	4☐	5☐
**D. C0TKNMEDURXWY**<br>In the past 30 days, how often have you taken your medications differently from how they are prescribed?	1☐	2☐	3☐	4☐	5☐
**E. C0THGTHURT**<br>In the past 30 days, how often have you seriously thought about hurting yourself?	1☐	2☐	3☐	4☐	5☐
**F. C0TMTHKOPI**	1☐	2☐	3☐	4☐	5☐

In the past 30 days, how much of your time was spent thinking about opioid medications (having enough, taking them, dosing schedule, etc.)?					
**G. C0ARGUE**<br>In the past 30 days, how often have you been in an argument?	1☐	2☐	3☐	4☐	5☐
**H. C0TRBCNTLANGER**<br>In the past 30 days, how often have you had trouble controlling your anger (e.g., road rage, screaming, etc.)?	1☐	2☐	3☐	4☐	5☐
**I. C0TKMEDSMO**<br>In the past 30 days, how often have you needed to take pain medications belonging to someone else?	1☐	2☐	3☐	4☐	5☐
**J. C0WRRHDLMED**<br>In the past 30 days, how often have you been worried about how you're handling your medications?	1☐	2☐	3☐	4☐	5☐
**K. C0OTHWRRHDLMED**<br>In the past 30 days, how often have others been worried about how you're handling your medications?	1☐	2☐	3☐	4☐	5☐
**L. C0ERCALL**<br>In the past 30 days, how often have you had to make an emergency phone call or show up at the clinic without an appointment?	1☐	2☐	3☐	4☐	5☐
**M. C0ANGRYOTH**<br>In the past 30 days, how often have you gotten angry with people?	1☐	2☐	3☐	4☐	5☐
**N. C0MOREMED**<br>In the past 30 days, how often have you had to take more of your medication than prescribed?	1☐	2☐	3☐	4☐	5☐
**O. C0BRRWPMED**<br>In the past 30 days, how often have you borrowed pain medication from someone else?	1☐	2☐	3☐	4☐	5☐
**P. C0PMEDOTHRSN**<br>In the past 30 days, how often have you used your pain medicine for symptoms other than for pain (e.g., to help you sleep, improve your mood, or relieve stress)?	1☐	2☐	3☐	4☐	5☐
**Q. C0VSTER**<br>In the past 30 days, how often have you had to visit the Emergency Room?	1☐	2☐	3☐	4☐	5☐

CHRONIC PAIN COPING INVENTORY - 42

The next series of questions ask you about non-medication self-management strategies for reducing chronic pain. *Self- management includes regular relaxation, physical activity, talking with others, and/or making time for social activities.*

57. During ***the past week***, how many days did you use each of the following at least once in the day to cope with your pain? (Note: You may have used some of these coping strategies on days that you did not have pain or to prevent or minimize pain in the future. Please indicate the number of days you used each strategy <u>for pain</u>, whether or not you were experiencing pain at the time).

Question	Number of Days
**A. P0RELAX**<br>Imagined a calming or distracting image to help me relax	☐0 ☐1 ☐2 ☐3 ☐4 ☐5 ☐6 ☐7

**B. P0IGNPAIN**<br>Ignored the pain	☐0 ☐1 ☐2 ☐3 ☐4 ☐5 ☐6 ☐7
**C. P0REST**<br>I took a rest	☐0 ☐1 ☐2 ☐3 ☐4 ☐5 ☐6 ☐7
**D. P0SUPTFRD**<br>I got support from a friend	☐0 ☐1 ☐2 ☐3 ☐4 ☐5 ☐6 ☐7
**E. P0ASKOTHHLP**<br>Asked someone to do something for me	☐0 ☐1 ☐2 ☐3 ☐4 ☐5 ☐6 ☐7
**F. P0WORSE**<br>Reminded myself that things could be worse	☐0 ☐1 ☐2 ☐3 ☐4 ☐5 ☐6 ☐7
**G. P0AVOID**<br>Avoided using part of my body (e.g. hand, arm, leg)	☐0 ☐1 ☐2 ☐3 ☐4 ☐5 ☐6 ☐7
**H. P0RELXMSL**<br>Focused on relaxing my muscles	☐0 ☐1 ☐2 ☐3 ☐4 ☐5 ☐6 ☐7
**I. P0STRTCH**<br>Sat on the floor ,stretched ,and held the stretch at least 10 seconds	☐0 ☐1 ☐2 ☐3 ☐4 ☐5 ☐6 ☐7
**J. P0BETTER**<br>Told myself things would get better	☐0 ☐1 ☐2 ☐3 ☐4 ☐5 ☐6 ☐7
**K. P0SPTFAM**<br>I got support from a family member	☐0 ☐1 ☐2 ☐3 ☐4 ☐5 ☐6 ☐7
**L. P0RESTMUCH**<br>I rested as much as I could	☐0 ☐1 ☐2 ☐3 ☐4 ☐5 ☐6 ☐7
**M. P0TALK**<br>I talked to someone close to me	☐0 ☐1 ☐2 ☐3 ☐4 ☐5 ☐6 ☐7
**N. P0CALLFRD**<br>Called a friend on the phone to help me feel better	☐0 ☐1 ☐2 ☐3 ☐4 ☐5 ☐6 ☐7
**O. P0THTGOOD**<br>Thought about all the good things I have	☐0 ☐1 ☐2 ☐3 ☐4 ☐5 ☐6 ☐7
**P. P0HLPTASK**<br>Asked for help with a chore or task	☐0 ☐1 ☐2 ☐3 ☐4 ☐5 ☐6 ☐7
**Q. P0PAINBET**<br>Told myself my pain would get better	☐0 ☐1 ☐2 ☐3 ☐4 ☐5 ☐6 ☐7
**R. P0NOINTFACT**<br>I didn't let the pain interfere with my activities	☐0 ☐1 ☐2 ☐3 ☐4 ☐5 ☐6 ☐7
**S. P0AERBEXE**<br>Engaged in aerobic exercise (exercise that made my heart beat faster) for at least 15 min.	☐0 ☐1 ☐2 ☐3 ☐4 ☐5 ☐6 ☐7
**T. P0LMTWLK**<br>Limited my walking because of pain	☐0 ☐1 ☐2 ☐3 ☐4 ☐5 ☐6 ☐7
**U. P0NPAPAIN**<br>Just didn't' pay attention to the pain	☐0 ☐1 ☐2 ☐3 ☐4 ☐5 ☐6 ☐7
**V. P0WLKLMP**<br>Walked with a limp to decrease the pain	☐0 ☐1 ☐2 ☐3 ☐4 ☐5 ☐6 ☐7
**W. P0MEDRELX**<br>Meditated to relax	☐0 ☐1 ☐2 ☐3 ☐4 ☐5 ☐6 ☐7
**X. P0LAYBACK**<br>Lay on my back, stretched, and held the stretch at least 10 seconds	☐0 ☐1 ☐2 ☐3 ☐4 ☐5 ☐6 ☐7
**Y. P0HLDPST**<br>Held part of my body (e.g. arm) in a special position	☐0 ☐1 ☐2 ☐3 ☐4 ☐5 ☐6 ☐7
**Z. P0HLPLIFT**<br>Asked for help in carrying, lifting or pushing something	☐0 ☐1 ☐2 ☐3 ☐4 ☐5 ☐6 ☐7
**AA. P0EXEIMPV**<br>Exercised to improve my overall physical condition for at least 5 minutes	☐0 ☐1 ☐2 ☐3 ☐4 ☐5 ☐6 ☐7
**BB. P0TLKSUPT**<br>Talked to a friend or family member for support	☐0 ☐1 ☐2 ☐3 ☐4 ☐5 ☐6 ☐7
**CC. P0RMDOTHWRS**	

Reminded myself that there are people who are worse off than I am	☐0 ☐1 ☐2 ☐3 ☐4 ☐5 ☐6 ☐7
**DD. P0LMTSTNDTM**<br>Limited my standing time	☐0 ☐1 ☐2 ☐3 ☐4 ☐5 ☐6 ☐7
**EE. P0LAYDWN**<br>Lay down on a bed	☐0 ☐1 ☐2 ☐3 ☐4 ☐5 ☐6 ☐7
**FF. P0AVDPHYACT**<br>Avoided some physical activities (lifting, pushing, carrying)	☐0 ☐1 ☐2 ☐3 ☐4 ☐5 ☐6 ☐7
**GG. P0SHRELAX**<br>Used self-hypnosis to relax	☐0 ☐1 ☐2 ☐3 ☐4 ☐5 ☐6 ☐7
**HH. P0KPTGO**<br>I just kept going	☐0 ☐1 ☐2 ☐3 ☐4 ☐5 ☐6 ☐7
**II. P0STRCHMSCL**<br>Stretched the muscles where I hurt and held the stretch for at least 10 seconds	☐0 ☐1 ☐2 ☐3 ☐4 ☐5 ☐6 ☐7
**JJ. P0AVDACT**<br>Avoided activity	☐0 ☐1 ☐2 ☐3 ☐4 ☐5 ☐6 ☐7
**KK. P0RMREST**<br>Went into a room by myself to rest	☐0 ☐1 ☐2 ☐3 ☐4 ☐5 ☐6 ☐7
**LL. P0DPBRTHRLX**<br>Used deep, slow breathing to relax	☐0 ☐1 ☐2 ☐3 ☐4 ☐5 ☐6 ☐7
**MM. P0EXEMSCLBK**<br>Exercised to strengthen the muscles in my back for at least 1 minute	☐0 ☐1 ☐2 ☐3 ☐4 ☐5 ☐6 ☐7
**NN. P0ASKGETSMTH**<br>Asked someone to get me something (e.g. medicine, food, drink)	☐0 ☐1 ☐2 ☐3 ☐4 ☐5 ☐6 ☐7
**OO. P0PAINOAFFCT**<br>Did not let the pain affect what I was doing	☐0 ☐1 ☐2 ☐3 ☐4 ☐5 ☐6 ☐7
**PP. P0LAYDWSOFA**<br>Lay down on a sofa	☐0 ☐1 ☐2 ☐3 ☐4 ☐5 ☐6 ☐7

FACIT-Sp-Ex (version 4)

58. Now I am going to ask you a few questions about your spirituality. Please indicate how much you agree with each statement by marking one box per row.

Statement	Not at all	A little bit	Somewhat	Quite a bit	Very much
**A. F0STRGFAITH**<br>I find strength in my faith or spiritual beliefs	1☐	2☐	3☐	4☐	5☐
**B. F0HIGHPW**<br>I feel connected to a higher power (or God)	1☐	2☐	3☐	4☐	5☐

PAIN SELF EFFICACY QUESTIONNAIRE (PSEQ)

59. Please rate how confident you are that you can do the following things at present, despite the pain. To indicate your answer circle one of the numbers on the scale under each item, where 0 = not at all confident and 6 = completely confident. Remember, we are not asking whether or not you have been doing these things, but rather how confident you are that you can do them at present, despite the pain.

Question	Not at all Confident					Completely Confident
**A. P0ENJOY**	0☐	1☐	2☐	3☐	4☐	5☐	6☐

I can enjoy things, despite the pain.							
**B. P0DOCHORE**<br>I can do most of the household chores (e.g. tidying-up, washing dishes, etc.), despite the pain.	0☐	1☐	2☐	3☐	4☐	5☐	6☐
**C. P0SOCFRND**<br>I can socialize with my friends or family members as often as I used to do, despite the pain.	0☐	1☐	2☐	3☐	4☐	5☐	6☐
**D. P0COPEPAIN**<br>I can cope with my pain in most situations.	0☐	1☐	2☐	3☐	4☐	5☐	6☐
**E. P0DOWORK**<br>I can do some form of work, despite the pain. ("work" includes housework, paid and unpaid work).	0☐	1☐	2☐	3☐	4☐	5☐	6☐
**F. P0DOMTHG**<br>I can still do many of the things I enjoy doing, such as hobbies or leisure activity, despite the pain.	0☐	1☐	2☐	3☐	4☐	5☐	6☐
**G. P0COPEWOMED**<br>I can cope with my pain without medication.	0☐	1☐	2☐	3☐	4☐	5☐	6☐
**H. P0ACCMPGOAL**<br>I can still accomplish most of my goals in life, despite the pain.	0☐	1☐	2☐	3☐	4☐	5☐	6☐
**I. P0LIVENMLF**<br>I can live a normal lifestyle, despite the pain.	0☐	1☐	2☐	3☐	4☐	5☐	6☐
**J. P0MOREACTV**<br>I can gradually become more active, despite the pain.	0☐	1☐	2☐	3☐	4☐	5☐	6☐

PATIENT-REPORTED OUTCOME MEASUREMENT INFORMATION SYSTEM 29-ITEM HEALTH PROFILE (PROMIS-29) AND SEXUAL FUNCTIONING

60. The next sets of questions ask you about your activity level, emotional well-begin, and pain level. Please respond to each question or statement by marking one box per row.

**Physical Function**	**Without any difficulty**	**With a little difficulty**	**With some difficulty**	**With much difficulty**	**Unable to do**
**A. P0CHORE**<br>Are you able to do chores such as vacuuming or yard work?	1☐	2☐	3☐	4☐	5☐
**B. P0STAIRS**<br>Are you able to go up and down stairs at a normal pace?	1☐	2☐	3☐	4☐	5☐
**C. P0WALK**<br>Are you able to go for a walk of at least 15 minutes?	1☐	2☐	3☐	4☐	5☐
**Anxiety**	**Never**	**Rarely**	**Sometimes**	**Often**	**Always**
In the past *7 **days***….					
**E. P0FEAR**<br>I felt fearful…..	1☐	2☐	3☐	4☐	5☐
**F. P0FOCUS**<br>I found it hard to focus on anything other than my anxiety…	1☐	2☐	3☐	4☐	5☐
**G. P0WORRY**<br>My worries overwhelmed me……	1☐	2☐	3☐	4☐	5☐
**H. P0UNEASY**<br>I felt uneasy……	1☐	2☐	3☐	4☐	5☐
**Depression**	**Never**	**Rarely**	**Sometimes**	**Often**	**Always**
In the past *7 **days***….					
**I. P0WRTHLS**<br>I felt worthless……	1☐	2☐	3☐	4☐	5☐

**J. P0HLPLS**<br>I felt helpless…….	1☐	2☐	3☐	4☐	5☐
**K. P0DEPRESS**<br>I felt depressed……	1☐	2☐	3☐	4☐	5☐
**L. P0HOPELS**<br>I felt hopeless…….	1☐	2☐	3☐	4☐	5☐
**Fatigue**	**Not at all**	**A little bit**	**Somewhat**	**Quite a bit**	**Very much**
During the past *7 **days***….					
**M. P0FLTFTG**<br>I felt fatigued……	1☐	2☐	3☐	4☐	5☐
**N. P0TRBTHK**<br>I have trouble starting thinking because I am tired……	1☐	2☐	3☐	4☐	5☐
In the past *7 **days***….					
**O. P0RUNDWN**<br>How run down did you feel on average?	1☐	2☐	3☐	4☐	5☐
**P. P0HOWFTG**<br>How fatigued were you on average?	1☐	2☐	3☐	4☐	5☐
**Sleep Disturbance**	**Very poor**	**Poor**	**Fair**	**Good**	**Very good**
In the past *7 **days***….					
**Q. P0SLPQLT**<br>My sleep quality was…..	1☐	2☐	3☐	4☐	5☐
In the past *7 **days***….	**Not at all**	**A little bit**	**Somewhat**	**Quite a bit**	**Very much**
**R. P0SLPRFRSH**<br>My sleep was refreshing…..	1☐	2☐	3☐	4☐	5☐
**S. P0PRBSLP**<br>I had a problem with my sleep…..	1☐	2☐	3☐	4☐	5☐
**T. P0DFTSLP**<br>I had difficulty falling asleep….	1☐	2☐	3☐	4☐	5☐
**Satisfaction with Social Role**	**Not at all**	**A little bit**	**Somewhat**	**Quite a bit**	**Very much**
In the past *7 **days***….					
U. **P0SATWRK**<br>I am satisfied with how much work I can do (include work at home)	1☐	2☐	3☐	4☐	5☐
**V. P0SATWKABLTY**<br>I am satisfied with my ability to work (include work at home)	1☐	2☐	3☐	4☐	5☐
**W. P0SATRESPNS**<br>I am satisfied with my ability to do regular personal and household responsibilities	1☐	2☐	3☐	4☐	5☐
**X. P0SATPERFM**<br>I am satisfied with my ability to perform my daily routines	1☐	2☐	3☐	4☐	5☐
**Pain Interference**	**Not at all**	**A little bit**	**Somewhat**	**Quite a bit**	**Very much**
In the past *7 **days***….					
**Y. P0PAINACT**<br>How much did pain interfere with your day to day activities?	1☐	2☐	3☐	4☐	5☐
**Z. P0PAINWRK**<br>How much did pain interfere with work around the home?	1☐	2☐	3☐	4☐	5☐
**AA. P0PAINSOC**<br>How much did pain interfere with your ability to participate in social activities?	1☐	2☐	3☐	4☐	5☐
**BB. P0PAINCHORE**	1☐	2☐	3☐	4☐	5☐

How much did pain interfere with your household chores?					
**Pain Intensity**					
In the past *7 days*….					

CC. P0PAINRT
How would you rate your pain on average on a scale of 1 to 10?

1 2 3 4 5 6 7 8 9 10

|-----------|----------|-----------|-----------|-----------|----------|-----------|----------|-----------|

No Pain **Worst Imaginable Pain**

Answer: _____

PROMIS

Next are just a few questions about your sex life.

61. P0SEXACT
In the past ***30 days…*** Have you had any type of sexual activity with another person (including your partner)?

1☐ Yes
2☐ No

62. P0STSSEXLF
In the past ***30 days…*** How satisfied have you been with your sex life?

1☐ Not at all
2☐ A little bit
3☐ Somewhat
4☐ Quite a bit
5☐ Very

SOCIAL SUPPORT

63. S0PSNHELP
How many people will help you with practical things when you need it, like giving you rides, helping with baby-sitting, a quick loan, and so forth?

______ number of people

64. S0FAMCNTCT
About how many family members (including spouse) and friends, lovers or partners would you say you regularly have contact with? (By regularly, we mean you see them or talk on the phone with them at least twice a month on average.)

______ number of people

65. S0PSNTALK
How many people do you have in your life to talk to when you worry about personal problems, such as family or work?

______ number of people

PATIENT HEALTH QUESTIONNAIRE-9 (PHQ-9)

66. Now I am going to ask you about your mood in the past two weeks.
Over the last 2 weeks, how often have you been bothered by any of the following problems?

	Not at all	Several days	More than half the days	Nearly every day
A. **P0LILPLS**<br>Little interest or pleasure in doing things	0☐	1☐	2☐	3☐
B. **P0DEPRES**<br>Feeling down, depressed, or hopeless	0☐	1☐	2☐	3☐
C. **P0TRBSLP**<br>Trouble falling or staying asleep, or sleeping too much	0☐	1☐	2☐	3☐
D. **P0TIRED**<br>Feeling tired or having little energy	0☐	1☐	2☐	3☐
E. **P0APPET**<br>Poor appetite or overeating	0☐	1☐	2☐	3☐
F. **P0FELBAD**<br>Feeling bad about yourself — or that you are a failure or have let yourself or your family down	0☐	1☐	2☐	3☐
G. **P0CONCEN**<br>Trouble concentrating on things, such as reading the newspaper or watching television	0☐	1☐	2☐	3☐
H. **P0SLOW**<br>Moving or speaking so slowly that other people could have noticed? Or the opposite — being so fidgety or restless that you have been moving around a lot more than usual	0☐	1☐	2☐	3☐
I. **P0HURT**<br>Thoughts that you would be better off dead or of hurting yourself in some way	0☐	1☐	2☐	3☐

J. **P0DIFWOR**<br>**IF P0LILPLS THRU P0HURT=0 THEN SKIP:**<br>If you checked off any problems, how difficult have these problems made it for you to do your work, take care of things at home, or get along with other people?	Not difficult at all<br>1☐	Somewhat difficult<br>2☐	Very difficult<br>3☐	Extremely difficult<br>4☐

PROMIS GLOBAL HEALTH (QoL)

The following are questions about your general quality of life.

67. Please respond to each item by marking one box per row.

Statement	Poor	Fair	Good	Very good	Excellent
A. **Q0HELTH**<br>In general, would you say your health is:	1☐	2☐	3☐	4☐	5☐
B. **Q0QLTLIFE**<br>In general, would you say your quality of life is:	1☐	2☐	3☐	4☐	5☐
C. **Q0RTPHLTH**<br>In general, how would you rate your physical health?	1☐	2☐	3☐	4☐	5☐
D. **Q0RTMHLTH**<br>In general, how would you rate your mental health, including your mood and your ability to think?	1☐	2☐	3☐	4☐	5☐
E. **Q0RTSTSSOC**<br>In general, how would you rate your satisfaction with your social activities and relationships?	1☐	2☐	3☐	4☐	5☐
F. **Q0RTSOCACT**<br>In general, please rate how well you carry out your usual social activities and roles. (This includes activities at home, at work and in your community, and responsibilities as a parent, child, spouse, employee, friend, etc.).	1☐	2☐	3☐	4☐	5☐
	**Not at all**	**A little**	**Moderately**	**Mostly**	**Completely**
G. **Q0PHYACT**<br>To what extent are you able to carry out your everyday physical activities such as walking, climbing stairs, carrying groceries, or moving a chair?	1☐	2☐	3☐	4☐	5☐
**IN THE PAST 7 DAYS….**	**Always**	**Often**	**Sometimes**	**Rarely**	**Never**
H. **Q0EMPRB**<br>How often have you been bothered by emotional problems such as feeling anxious, depressed or irritable?	1☐	2☐	3☐	4☐	5☐
I. **Q0RTFTG**<br>How would you rate your fatigue on average?	**Very severe**	**Severe**	**Moderate**	**Mild**	**None**
	1☐	2☐	3☐	4☐	5☐

SUBSTANCE USE QUESTION NIDA-MODIFIED ASSIST

Next we are going to ask you some questions about substance use.
As a reminder, your answers are completely confidential.

68. In your *LIFETIME*, which of the following substances have you EVER used?

Question	Yes	No
**A. A0EVEMAR**<br>**Cannabis** (marijuana, pot, grass, hash, etc.)	1☐	2☐
**B. A0EVECOC**<br>**Cocaine** (coke, crack, etc.)	1☐	2☐
**C. A0EVERXSTI**<br>**Prescription stimulants** (Ritalin, Concerta, Dexedrine, Adderall, diet pills, etc.)	1☐	2☐
**D. A0EVEMET**<br>**Methamphetamine** (speed, crystal meth, ice, etc.)	1☐	2☐
**E. A0EVEINH**<br>**Inhalants** (nitrous oxide, glue, gas, paint thinner, etc.)	1☐	2☐
**F. A0EVESED**<br>**Sedatives or sleeping pills** (Valium, Serepax, Ativan, Xanax, Librium, Rohypnol, GHB, etc.)	1☐	2☐
**G. A0EVEHAL**<br>**Hallucinogens** (LSD, acid, mushrooms, PCP, Special K, ecstasy, etc.)	1☐	2☐
**H. A0EVEHER**<br>**Street opioids** (heroin, opium, etc.)	1☐	2☐
**I. A0EVERXOPI**<br>**Prescription opioids** (fentanyl, oxycodone [OxyContin, Percocet], hydrocodone [Vicodin], methadone, buprenorphine, etc.)	1☐	2☐
**J. A0EVEOTH**<br>**Other – Specify:**	1☐	2☐

IF **A0EVEMAR THRU A0EVEOTH = 1 THEN GO TO A0USEDRG3M**
IF **A0EVEMAR THRU A0EVEOTH = 2 THEN SKIP TO S0SMK100**

69. Ask the following questions for each drug mentioned above:

Question	Never	Once or Twice	Monthly	Weekly	Daily or Almost Daily
**A. A0USEDRG3M**<br>*In the past 3 months*, how often have you used (insert name of drug)?<br>IF **A0USEDRG3M = 1 THEN SKIP TO A0FRDCRNDRG**	1☐	2☐	3☐	4☐	5☐
**B. A0DESRUSEDRG**<br>In the past 3 months, how often have you had a strong desire or urge to use (insert name of drug)?	1☐	2☐	3☐	4☐	5☐
**C. A0OFTUSEDRG**<br>In the past 3 months, how often has your use of (insert name of drug) led to	1☐	2☐	3☐	4☐	5☐

health, social, legal or financial problems?					
**D. A0FAILDO** In the past 3 months, how often have you failed to do what was normally expected of you because of your use of (insert name of drug)?	1☐	2☐	3☐	4☐	5☐

Ask Questions "E"& "F" for all substances *ever used*:	**NO**	**YES**, but not in the last 3 months	**YES**, in the past three months
**E. A0FRDCRNDRG** Has a friend or relative or anyone else ever expressed concern about your use of (insert name of drug)?	1☐	2☐	3☐
**F. A0FAILCUTDRG** Have you ever tried and failed to control, cut down, or stop using (insert name of drug)?	1☐	2☐	3☐

IF A0EVEMAR =1, AND A0USEDRG3M_MAR = 2,3,4,5, THEN A0MAR30D …

(IF A0EVEMAR = 2 OR A0USEDRG3M_MAR = 1 THEN SKIP TO S0SMK100)

70. **A0MAR30D**
Did you use marijuana in past 30 days for medical purposes, as prescribed by a health care provider?

1☐ Yes
2☐ No
3☐ Sometimes for medical reasons, sometimes not
97 ☐ Don't Know/Refused

70A. **A0MARRSN**
Please indicate all the health or medical reasons for which you used marijuana in the past 30 days.

1☐ Pain
2☐ Appetite
3☐ Anxiety
4☐ Depression
5☐ Sleep
7☐ Other (Specify): ___________________
97 ☐ Don't Know/Refused

CIGARETTES/SMOKING

Now we are going to ask you a few questions about smoking.

71. S0SMK100
Have you smoked at least 100 cigarettes (5 packs or more) in your lifetime?
1☐ Yes
2☐ No →**SKIP TO Q76**

72. **S0SMKFSTAGE**
How old were you when you first smoked cigarettes regularly?
___ ___ Years Old

73. **S0SMKAVGDY**
On average, how many cigarettes per day do you usually smoke (or did you usually smoke when you did smoke)?
___ ___ # of Cigarettes

74. **S0DYSMK30D**
In the past 30 days, on how many days did you smoke at least one cigarette?
___ ___# Days

75. **S0SMKLSTAGE**
How old were you when you last smoked cigarettes regularly?
___ ___ Years Old

76. **S0EVECIG**
Have you ever used Electronic Cigarettes or other forms of Electronic Nicotine Delivery Systems (ENDS) such as E-Hookah or Vape Pens?

1☐ No **→SKIP TO T0ALCM5 or T0ALCF4**
2☐ Yes, More than a year ago, but not in the past year **→SKIP TO T0ALCM5 or T0ALCF4**
3☐ Yes, In the past year, but more than a month ago **→SKIP TO T0ALCM5 or T0ALCF4**
4☐ Yes, In the past month

77. **S0ECIG30D**
In the past 30 days, on how many days did you use an E-cigarette/E-Hookah/Vape pen?
___ ___ # of Days

ALCOHOL USE

1 drink is equal to a 12 ounce can of beer, a 5 ounce glass of wine, a 1.5 ounce shot of liquor, or an 8-9 ounce malt liquor

IF D0GENDER ≠ 1 OR (D0GENDER = 1 AND D0AGE > 65) THEN SKIP TO T0ALCF5

78. T0ALCM5
How many times in the past three months have you had 5+ drinks containing alcohol in a day?
___ ___ # of Days

IF D0GENDER = 1 AND D0AGE <= 65 THEN SKIP TO T0ALC1WK

79. T0ALCF4
How many times in the past three months have you had 4+ drinks containing alcohol in a day?
___ ___ # of Days

80. T0DRINKS
On a typical drinking day, how many drinks do you have?
___ ___ # of Drinks

81. T0ALC1WK
On average, how many days a week do you have an alcoholic drink?
___ ___ # of Days

IF MEN,
AND >4 DRINKS PER DAY (**T0DRINKS** > 4), OR
>14 DRINKS PER WEEK (**T0DRINKS** * **T0ALC1WK**), THEN ASK THE FOLLOWING TWO QUESTIONS

82. T0HURTMALC
In the past year, have you sometimes been under the influence of alcohol in situations where you could have caused an accident or gotten hurt?
1☐ Yes
2☐ No

83. T0MOREMALC
Have there often been times when you had a lot more to drink than you intended to have?
1☐ Yes
2☐ No

IF MEN>=65 OR WOMEN,
AND > 3 DRINKS PER DAY (**T0DRINKS** > 3), OR
> 7 DRINKS PER WEEK (**T0DRINKS** * **T0ALC1WK**), THEN ASK THE FOLLOWING TWO QUESTIONS

84. T0HURTFALC
In the past year, have you sometimes been under the influence of alcohol in situations where you could have caused an accident or gotten hurt?
1☐ Yes

2☐ No

85. T0MOREFALC

Have there often been times when you had a lot more to drink than you intended to have?

1☐ Yes

2☐ No

86. H0PCPTLK

Have you ever talked to your Primary Care Provider about drug or alcohol problems?

1☐ Yes

2☐ No **→SKIP TO Q88**

97 ☐ Don't Know/Refused

87. H0SPKDOC

How often in the past year have you spoken with your Primary Care Provider about this?

1☐ Once

2☐ More than once

ADVERSE CHILDHOOD EXPERIENCE (ACE)

While you were growing up, during your first 18 years of life:

88. A0INSULT
Did a parent or other adult in the household **often or very often**…
Swear at you, insult you, put you down, or humiliate you? **OR**
Act in a way that made you afraid that you might be physically hurt?
1☐ Yes
2☐ No
97☐ Refused/Don't know

89. A0HIT
Did a parent or other adult in the household **often or very often**…
Push, grab, slap, or throw something at you? **OR**
Ever hit you so hard that you had marks or were injured?
1☐ Yes
2☐ No
97☐ Refused/Don't know

90. A0ATTMPSEX
Did an adult or person at least 5 years older than you **ever**…
Touch or fondle you or have you touch their body in a sexual way? **OR**
Attempt or actually have oral, anal, or vaginal intercourse with you?
1☐ Yes
2☐ No
97☐ Refused/Don't know

91. A0NOLOVE
Did you **often or very often** feel that …
No one in your family loved you or thought you were important or special? **OR**
Your family didn't look out for each other, feel close to each other, or support each other?
1☐ Yes
2☐ No
97☐ Refused/Don't know

92. A0NOCARE
Did you **often or very often** feel that …
You didn't have enough to eat, had to wear dirty clothes, and had no one to protect you? **OR**
Your parents were too drunk or high to take care of you or take you to the doctor if you needed it?
1☐ Yes
2☐ No
97☐ Refused/Don't know

93. A0PARNTDVC
Were your parents **ever** separated or divorced?
1☐ Yes
2☐ No
97☐ Refused/Don't know

94. A0MOMPUSH

Was your mother or stepmother:
Often or very often pushed, grabbed, slapped, or had something thrown at her? **OR**
Sometimes, often, or very often kicked, bitten, hit with a fist, or hit with something hard? **OR**
Ever repeatedly hit at least a few minutes or threatened with a gun or knife?

1☐ Yes
2☐ No
97☐ Refused/Don't know

95. A0HSMEMBSBPRB

Did you live with anyone who was a problem drinker or alcoholic or who used street drugs?

1☐ Yes
2☐ No
97☐ Refused/Don't know

96. A0HSMEMBDEP

Was a household member depressed or mentally ill, or did a household member attempt suicide?

1☐ Yes
2☐ No
97☐ Refused/Don't know

97. A0HSMEMBPRSN

Did a household member go to prison?

1☐ Yes
2☐ No
97☐ Refused/Don't know

PATIENT PROVIDER COMMUNICATION
Communication Assessment Tool

We would like to know how you feel about the way your primary care physician has communicated with you in your last couple of visits. Your answers are completely confidential, so please be as open and honest as you can.

98. Please rate your physician's communication with you by marking **one box per row.**

Statement	Poor	Fair	Good	Very good	Excellent
**My doctor**					
**A. C0GREET** Greeted me in a way that made me feel comfortable	1☐	2☐	3☐	4☐	5☐
**B. C0TXRSPT** Treated me with respect	1☐	2☐	3☐	4☐	5☐
**C. C0INSTIDEA** Showed interest in my ideas about my health	1☐	2☐	3☐	4☐	5☐
**D. C0UNDHLTCNR** Understood my main health concerns	1☐	2☐	3☐	4☐	5☐
**E. C0ATTEN** Paid attention to me (looked at me, listened carefully)	1☐	2☐	3☐	4☐	5☐
**F. C0NOINTRPT** Let me talk without interruptions	1☐	2☐	3☐	4☐	5☐
**G. C0GVINFO** Gave me as much information as I wanted	1☐	2☐	3☐	4☐	5☐
**H. C0TLKUNDSTD** Talked in terms I could understand	1☐	2☐	3☐	4☐	5☐
**I. C0CHKUNDSTD** Checked to be sure I understood everything	1☐	2☐	3☐	4☐	5☐
**J. C0ENCGASKQ** Encouraged me to ask questions	1☐	2☐	3☐	4☐	5☐
**K. C0INVDEC** Involved me in decisions as much as I wanted	1☐	2☐	3☐	4☐	5☐
**L. C0DISFU** Discussed next steps, including any follow-up plans	1☐	2☐	3☐	4☐	5☐
**M. C0CARECNR** Showed care and concern	1☐	2☐	3☐	4☐	5☐
**N. C0SPTTIME** Spent the right amount of time with me	1☐	2☐	3☐	4☐	5☐

PEPPI   Perceived Efficacy in Patient-Physician Interactions Questionnaire

99. Next, please rate your confidence level in your communication with your physician by marking one box per row.

Statement	Not at all confident				Very Confident
**How confident are you in your ability to:**					
**A. P0DRATTN**<br>Get a doctor to pay attention to what you have to say?	1☐	2☐	3☐	4☐	5☐
**B. P0KNWASKDC**<br>Know what questions to ask a doctor?	1☐	2☐	3☐	4☐	5☐
**C. P0DRANSQST**<br>Get a doctor to answer all of your questions?	1☐	2☐	3☐	4☐	5☐
**D. P0ASKDRHLTH**<br>Ask a doctor questions about your chief health concern?	1☐	2☐	3☐	4☐	5☐
**E. P0VSMSTTDR**<br>Make the most of your visit with the doctor?	1☐	2☐	3☐	4☐	5☐
**F. P0DRSERHLTH**<br>Get a doctor to take your chief health concerns seriously?	1☐	2☐	3☐	4☐	5☐
**G. P0UNDSTDR**<br>Understand what a doctor tells you?	1☐	2☐	3☐	4☐	5☐
**H. P0GTDRDOSM**<br>Get a doctor to do something about your chief health concern?	1☐	2☐	3☐	4☐	5☐
**I. P0EXPDRHLTHCRN**<br>Explain your chief health concern to a doctor?	1☐	2☐	3☐	4☐	5☐
**J. P0ASKMINF**<br>Ask a doctor for more information if you don't understand what he or she said?	1☐	2☐	3☐	4☐	5☐

You have completed the study baseline questionnaire.

Thank you!

List of common prescription opiate medications

Buprenorphine
Buprenorphine/Naloxone
Butalbital with Codeine
Butrans
Codeine
Darvocet
Darvon
Dihydrocodeine
Duragesic (Fentanyl transdermal)
Fentanyl
Fiorinal with codeine
Hydrocodone
Kadian
Levorphanol
Meperidine or Demerol
Methadone
Morphine
MS Contin
Norco
Nubain
Opana
Oramorph
Oxycodone
Oxycontin
OxyFast OxyIR
Oxymorphone
Pentazocine
Percocet
Propoxyphene
Reprexain
Roxanol
Roxicodone
Suboxone
Talwin
Tramadol or Ultram
Tylenol #3
Tylox
Vicodin
Vicoprofen
Zubsolv
