# Supplementary material for: A patient activation intervention in primary care for patients with chronic pain on long term opioid therapy: results from a randomized control trial
Source: BMC Health Serv Res. 2024 Jan 22;24:112. doi: 10.1186/s12913-024-10558-3 (PMC10802020; doi:10.1186/s12913-024-10558-3)
Supplement: Supplementary file 3 — Additional file 3. ACTIVATE study 12-month questionnaire. Interview-administered survey completed over telephone at 12-months post-enrollment. [file 12913_2024_10558_MOESM3_ESM.pdf]

ACTIVATE: Patients Managing Pain
12-month follow-up telephone interview

ENTERED BY RESEARCH STAFF BEFORE INTERVIEW BEGINS:

PPN: ___ ___ ___ ___ ___

Thank you again for agreeing to be part of the ACTIVATE Study. Please answer questions as honestly as you can. Your answers are confidential. You can refuse to answer any questions or stop the questionnaire at any time. We will be asking a variety of questions with various different time periods. Please refer to the time period specific to each question. Some of the questions may seem similar; please be assured every question is important.

DEMOGRAPHICS

1. **D2GENDER** – Gender [PRE-POPULATE FROM BASELINE]
1☐ Male
2☐ Female
3☐ Other (e.g., Transgender, Intersex)
Specify: ___________________

2. **D2MASTAT** – Marital status
What is your current marital status?
1☐ Married
2☐ Living with Intimate Partner
3☐ Widowed
4☐ Divorced or separated
5☐ Single, never married

3. **D2EMSTAT** - Employment Status
What best describes your current employment status?
1☐ Employed full-time
2☐ Employed part-time
3☐ Unemployed
4☐ Retired
5☐ Fulltime Homemaker **→SKIP TO D2EDUC**
6☐ Student (Not employed) **→ SKIP TO D2EDUC**
7☐ Disabled

4. **D2EDUC** – Education
What is the highest level of education you have completed?
1☐ 12 years or less: ___ ___
2☐ High School diploma or GED
3☐ A.A., A.S., other vocational program
4☐ B.A., B.S.
5☐ M.A., M.S.
6☐ Ph.D., M.D., J.D.

5. **D2LIVSIT** – Current living situation
What best describes your current living situation?
Check the box that best applies

1☐ House or apartment (you rent or own)
2☐ House or apartment of friend or relative
3☐ Halfway house or residence in therapeutic community
4☐ Single room occupancy (SRO), hotel or motel
5☐ Homeless (streets, shelter, car, no stable arrangement)
6☐ Institution (jail, hospital)
7☐ Other (Specify): ___________________

6. **D2LIVSUP** – Current living support
Who do you currently live with?
1☐ Alone
2☐ With friend(s) or relative(s)
7☐ Other (Specify): ___________________

7. **D2INCOME**
What is your yearly household income after taxes?
Check the box that applies

1☐ Less than $10,000
2☐ $10,001 - $20,000
3☐ $20,001 - $30,000
4☐ $30,001 - $40,000
5☐ $40,001 - $50,000
6☐ $50,001 - $60,000
7☐ $60,001 - $70,000
8☐ $70,001 - $80,000
9☐ $80,001 - $90,000
10☐ $90,001 - $100,000
11☐ over $100,000
97 ☐ Rather not say

PATIENT ACTIVATION (PAM)

Now we will ask you some questions about how you feel about your involvement in your own healthcare.

8. Below are some statements that people sometimes make when they talk about their health. Please indicate how much you agree or disagree with each statement as it applies to you personally by checking your answer.

Question	Disagree Strongly	Disagree	Agree	Strongly Agree
**A. P2HLTHRESP**<br>When all is said and done, I am the person who is responsible for taking care of my health	1☐	2☐	3☐	4☐
**B. P2HTLTH ACT**<br>Taking an active role in my own health care is the most important thing that affects my health	1☐	2☐	3☐	4☐
**C. P2HLTH CON**<br>I am confident I can help prevent or reduce problems associated with my health	1☐	2☐	3☐	4☐
**D. P2KNOWRX**<br>I know what each of my prescribed medications do	1☐	2☐	3☐	4☐
**E. P2GODOC**<br>I am confident that I can tell whether I need to go to the doctor or whether I can take care of a health problem myself	1☐	2☐	3☐	4☐
**F. P2TELLDOC**<br>I am confident that I can tell a doctor concerns I have even when he or she does not ask	1☐	2☐	3☐	4☐

**G. P2CONFTX**<br>I am confident that I can follow through on medical treatments I may need to do at home	1☐	2☐	3☐	4☐
**H. P2UNDER**<br>I understand my health problems and what causes them	1☐	2☐	3☐	4☐
**I. P2TXAVAIL**<br>I know what treatments are available for my health problems	1☐	2☐	3☐	4☐
**J. P2MAIN**<br>I have been able to maintain (keep up with) lifestyle changes, like eating right or exercising	1☐	2☐	3☐	4☐
**K. P2PREV**<br>I know how to prevent problems with my health	1☐	2☐	3☐	4☐
**L. P2SOLU**<br>I am confident I can figure out solutions when new problems arise with my health	1☐	2☐	3☐	4☐
**M. P2LIFE**<br>I am confident that I can maintain lifestyle changes, like eating right and exercising, even during times of stress	1☐	2☐	3☐	4☐

SATISFACTION WITH CARE

Now I will ask you some questions about your satisfaction with your health care.

9. **T2STSOVCARE**

How would you rate your satisfaction with your <u>overall health care</u> on a scale of "1" to "10," where "1" is the worst possible care and "10" is the best possible care?

1 2 3 4 5 6 7 8 9 10

|-----------|----------|-----------|-----------|-----------|----------|-----------|----------|-----------|

Worst Possible Care Best Possible Care

Answer: _____

10. **T2STSPCP**

How would you rate your satisfaction with your <u>primary care physician</u> on a scale of "1" to "10," where "1" is the worst possible care and "10" is the best possible care?

1 2 3 4 5 6 7 8 9 10

|-----------|----------|-----------|-----------|-----------|----------|-----------|----------|-----------|

Worst Possible Care Best Possible Care

Answer: _____

11. **T2STSPAINMG**

How would you rate your satisfaction with your <u>pain management care</u> on a scale of "1" to "10," where "1" is the worst possible care and "10" is the best possible care?

1 2 3 4 5 6 7 8 9 10

|-----------|----------|-----------|-----------|-----------|----------|-----------|----------|-----------|

Worst Possible Care Best Possible Care

Answer: _____

HEALTH CARE UTILIZATION

The next series of questions ask about your health care use. Please respond to the questions to the best of your ability.

12. **H2KPMEMB**
Are you still a Kaiser member?

1☐ Yes **IF YES, SKIP TO H2KPCOUNS**
2☐ No
97☐ Refused/Don't know

13. **H2NOKPRSN**
Why did you leave Kaiser Health Plan?

1☐ Moved out of area
2☐ Switched to another health plan
3☐ Lost job/lost coverage
4☐ Other ______________________________
97☐ Refused/Don't know

14. H2KPCOUNS
In the ***past 6 months,*** did you receive advice or counseling from a Kaiser doctor, nurse, health educator, or other Kaiser health care professional about the following? (This does not include appointments or group sessions with the study research staff or clinician.): *Check all that apply*

1☐ Pain management
2☐ Your diet (what you eat)
3☐ Losing weight
4☐ Getting more exercise
5☐ Quitting smoking
6☐ Stress or emotional problems (like depression)
7☐ Health screening tests recommended for you
8☐ No advice or counseling

15. H2KPPAINPGM
In the *past 6 months*, did you participate in a pain management program at Kaiser? (This does not include appointments or group sessions with the study research staff or clinician.)

1☐ Yes, attended
2☐ Was referred but never attended
3☐ No

16. H2COMP
Do you have access to a personal computer?
1☐ Yes, at home
2☐ Yes, at other location
3☐ No

17. H2INTER
Do you have access to the Internet?
1☐ Yes, at home
2☐ Yes, at other location
3☐ No

18. H2KPORG

Have you accessed KP.org in the past 6 months? (This does not include appointments or group sessions with the study research staff or clinician.)

1☐ Yes
2☐ No →**SKIP TO H2HLTHEDKP**
97☐ Don't Know/Refused

19. H2WAYSKP

In what ways have you used KP.org in the past 6 months**?** (This does not include appointments or group sessions with the study research staff or clinician.)

Check all that apply

1☐ Emailed my doctor or received an email from my doctor
2☐ Checked my lab results or immunization history
3☐ Scheduled or cancelled a medical appointment
4☐ Ordered a prescription refill
5☐ Reviewed information from a past visit
6☐ Used KP.org's Health and Wellness Resources (healthy lifestyle programs, wellness coaching, audio podcasts, recipe blogs, tools/calculators, videos).

20. H2HLTHEDKP

Have you attended a Kaiser Health Education Class in the past 6 months? (This does not include group sessions with the study clinician.)

1☐ Yes
2☐ No
97☐ Don't Know/Refused

The next several questions ask about health services you may have received OUTSIDE of Kaiser Permanente.

21. H2NONKPS

Excluding dental care, prescription refills or optometry/eyeglasses**,** did you receive any medical care **not paid for by** Kaiser Permanente during the past 6 months?

1☐ Yes
2☐ No →**SKIP TO C2TXPAINFR**

22. H2ERD1

During the past 6 months, how many days were you a patient in a non-Kaiser **emergency room** that Kaiser did not pay for?

___ ___ ___ Days

23. H2HOSPD1

During the past 6 months, how many days were you a patient **overnight (excluding for labor and delivery) in a non-Kaiser hospital** that Kaiser did not pay for?

___ ___ ___ Days

24. H2SAINV1

During the past 6 months, how many episodes of treatment did you have in an outpatient treatment program (NOT including AA or any other self-help group) for **alcohol or drug problems** outside of Kaiser that Kaiser did not pay for?

___ ___ ___ Times

25. **H2PAINTXNKP**
During the past 6 months, how many episodes of treatment in a **pain management** program outside of Kaiser did you have that Kaiser did not pay for?
___ ___ ___ Times

26. **H2SYMDV1**
During the past 6 months, how many times did you see a provider (e.g., psychiatrist, psychologist, social worker) for **mental health or emotional problems** as an outpatient outside of Kaiser that Kaiser did not pay for?
___ ___ ___ Times

27. **H2PCV1**
During the past 6 months, how many times did you see a **primary care provider** (nurse or physician) as an **outpatient** outside of Kaiser that Kaiser did not pay for?
___ ___ ___ Times

28. **H2OTHPV1**
During the past 6 months, how many times did you see **a medical provider in other clinics (e.g., dermatology or allergies)** as an outpatient outside of Kaiser that Kaiser did not pay for?
___ ___ ___ Times

CHRONIC PAIN EXPECTATIONS

We are interested in your expectations for relief of your pain. Please indicate how much you agree or disagree with each statement.

Question	Disagree Strongly	Disagree	Agree	Strongly Agree
29. **C2TXPAINFR**<br>I expect that treatment will make me pain free.	1☐	2☐	3☐	4☐
30. **C2ACCPPNTX**<br>I accept that I will have some pain with even the best treatment.	1☐	2☐	3☐	4☐

The next questions are about your use of prescription opioid pain medicines.

31. **C2OPIRXMED**
Are you **currently** taking prescription opioid pain medication? Some examples of opiate pain medicines are: Codeine, Darvocet, Darvon, Fentanyl, Hydrocodone, Methadone, Morphine, Norco, Oxycodone, Oxycontin, Percocet, Percodan, Propoxyphene, and Vicodin)
1☐ Yes
2☐ No SKIP TO **C2STPOPIRX**

32. **C2PAINMED**
What prescription opioid pain medications have you taken in the past two weeks?
Check all that apply USE DROP DOWN LIST FROM BASELINE

33. **C2PMEDDAYS**
In the past 2 weeks, about how many days did you take prescription opioid pain medicines?
_____ Days ENTER "0" IF DIDN'T TAKE OPIOIDS IN PAST 2 WEEKS
97☐ Refused/Don't know

34. **C2TMPMED**
In the past 2 weeks, about how many times per day did you usually take prescription opioid pain medicine on the days you used these medicines?
__ __ Times per day ENTER "0" IF DIDN'T TAKE OPIOIDS IN PAST 2 WEEKS
97☐ Refused/Don't know

35. **C2PMEDRX**
Do you always take your prescription opioid pain medication as prescribed?
1☐ Yes SKIP TO **C2PMADSE**
2☐ No

36. **C2NOPMEDRSN**
Please describe why you sometimes don't take your prescription opioid medication as prescribed.
Please check all that apply:
1☐ Side Effects
2☐ Poor pain control (take more than prescribed because amount prescribed doesn't help)
3☐ Do not need it
4☐ Concern about overuse
5☐ Pain is bad and need more than prescribed
7☐ Other (Specify): __________________

37. **C2PMADSE**
Considering all of the possible side effects of opiate medicines you may have experienced in the past month, (such as nausea, constipation, grogginess), how bothersome were these side effects?
1☐ Not at all bothersome
2☐ A little bothersome
3☐ Moderately bothersome
4☐ Very bothersome
5☐ Extremely bothersome
97☐ Refused/Don't know

Which two side effects bother you the most?
37A. C2SIDEFMOST1
Feeling groggy or sluggish
Feeling unstable or dizzy
Feeling confused or disoriented
Constipation
Nausea/vomiting
Nightmares
Itchiness
Difficulty urinating or difficulty with urinary retention
Less interest in sex or other sexual difficulties
Other: specify____________

37B **C2SIDEFMOST2**

Feeling groggy or sluggish
Feeling unstable or dizzy
Feeling confused or disoriented
Constipation
Nausea/vomiting
Nightmares
Itchiness
Difficulty urinating or difficulty with urinary retention
Less interest in sex or other sexual difficulties
Other: specify____________

38. **C2HELPOP**

Over the past month, how helpful have you found opiate pain medicines in relieving your pain.

1☐ Not at all helpful
2☐ A little helpful
3☐ Moderately helpful
4☐ Very helpful
5☐ Extremely helpful
97☐ Refused/Don't know

39. **C2IMPCUTMED**

How important is it for you to decrease your opioid use?

1☐ Not at all important
2☐ A little important
3☐ Moderately important
4☐ Very important
5☐ Extremely important
97☐ Refused/Don't know

40. **C2CONFCUTMED**

How confident are you that you can decrease your opioid use?

1☐ Not at all confident
2☐ A little confident
3☐ Moderately confident
4☐ Very confident
5☐ Extremely confident
97☐ Refused/Don't know

41. **C2LTMGOAL**

What is your **current** long-term goal for using prescription opioids for pain management?

1☐ Stay the same
2☐ Increase use
3☐ Decrease use
4☐ Stop use

42. **C2IMPGOAL**
How important is it for you to reach your goal?
1☐ Not at all important
2☐ A little important
3☐ Moderately important
4☐ Very important
5☐ Extremely important
97☐ Refused/Don't know

43. **C2DOCSTOP**
Has your doctor talked to you about stopping or tapering your opioids in the last 6 months?
1☐ Yes
2☐ No

44. **C2CUTOPIUSE**
Are you currently in the process of tapering (gradually decreasing) your opioid use?
1☐ Yes
2☐ No → skip to **C2GETOPINKP**

45. **C2DECPVDOPICUT**
Do you feel like a partner with your doctor in this decision to taper your opioid use?
1☐ Yes
2☐ No

NEXT THREE QUESTIONS ARE ONLY FOR THOSE NO LONGER TAKING OPIOIDS

46. **C2STPOPIRX**
How long ago did you stop taking opioids?

_______ months

47. **C2NOOPIRXRS**
Why did you stop taking opioids?

1☐ No longer needed
2☐ Too expensive
3☐ Side effects
4☐ Found other ways to decrease pain
5☐ Doctor strongly encouraged
6☐ Family encouraged
7☐ Other. Specify: ______________________

48. **C2DECPVDOPICUTNOOP**
Did you feel like a partner with your doctor in this decision to stop your opioid use?
1☐ Yes
2☐ No

NEXT 7 QUESTIONS ARE ASKED OF EVERYONE

49. **C2GETOPINKP**
Have you tried to obtain opioids from other sources in the last 6 months as result of your KP doctor wanting to decrease your opioid use?
1☐ Yes
2☐ No

50. **C2CHGDOCOPICUT**
Have you switched doctors in the last 6 months, as result of your KP doctor wanting to decrease your opioid use?
1☐ Yes
2☐ No

51. **C2LFKPOPICUT**
Have you thought about leaving Kaiser in the last 6 months, as result of your KP doctor wanting to decrease your opioid use?
1☐ Yes
2☐ No
3☐ NA – not a KP member in the last 6 months

Six months ago, you indicated your long tem goal for opioid use was to [INSERT ANSWER FROM **C1LTMGOAL**].

52. **C2OPIGOAL**
To what extent do you feel you have met your goals for opioid use in the past 6 months?
1☐ To a great extent → SKIP TO **C2MDREF**
2☐ Somewhat → SKIP TO **C2MDREF**
3☐ Very little
4☐ Not at all

53. **C2NOSUCCSS**
Why do you think you were not as successful as you would have liked?
1☐ Didn't have the support from doctor/health care system
2☐ Didn't have the support of family/friends
` 3☐ Pain is too bad
4☐ Unable to find alternative ways to reduce pain
5☐ Lost health insurance
6☐ Other: specify________________________

54. **C2MDREF**
How do you currently manage your pain? *Check all that apply*
1☐ Opioid medication prescribed by a doctor
2☐ Non-opioid medication prescribed by a doctor
3☐ Over the counter medication (like Tylenol)
2☐ Complementary/Alternative Medicine (i.e., acupuncture, herbs) Specify:____
3☐ Meditation, relaxation, or mindfulness practice
4☐ Pain classes or therapy (group or individual)
5☐ Massage or other bodywork
6☐ Exercise, stretching or physical therapy
7☐ Nothing
8☐ Other Specify:____
97☐ Refused/Don't know

55. **C2RXS**

During the <u>last 6 months,</u> how many of your own opiate prescriptions did you get filled at **NON-Kaiser** pharmacies (including through **NON-Kaiser** web sites)?

___ ___ ___ Prescriptions

[IF NO LONGER ON OPIOIDS (C2OPIRXMED = 2), SKIP the SOAPP and the COMM]

SCREENER AND OPIOID ASSESSMENT FOR PATIENTS WITH PAIN (SOAPP-5)

56. Now a few general questions about your mood and drug use.

Question	Never	Seldom	Sometimes	Often	Very Often
A. **S2MOODSWG**<br>How often do you have mood swings?	1☐	2☐	3☐	4☐	5☐
B. **S2SMKWP**<br>How often do you smoke a cigarette within an hour after you wake up?	1☐	2☐	3☐	4☐	5☐
C. **S2MEDNFRX**<br>How often have you taken medication other than the way that it was prescribed?	1☐	2☐	3☐	4☐	5☐
D. **S2USEDRG**<br>How often have you used illegal drugs (for example, marijuana, cocaine, etc.) in the past five years?	1☐	2☐	3☐	4☐	5☐
E. **S2LEGPRB**<br>How often, in your lifetime, have you had legal problems or been arrested?	1☐	2☐	3☐	4☐	5☐

CURRENT OPIOID MISUSE (COMM)

57. Please answer each question as honestly as possible. Keep in mind that we are only asking about the **PAST 30 DAYS**. There are no right or wrong answers. If you are unsure about how to answer the question, please give the best answer you can.

Question	Never	Seldom	Sometimes	Often	Very Often
**A. C2TRBMEMB**<br>In the past 30 days, how often have you had trouble with thinking clearly or had memory problems?	1☐	2☐	3☐	4☐	5☐
**B. C2UNDNTASK**<br>In the past 30 days, how often do people complain that you are not completing necessary tasks? (i.e., doing things that need to be done, such as going to class, work or appointments)	1☐	2☐	3☐	4☐	5☐
**C. C2GTNRXMED**<br>In the past 30 days, how often have you had to go to someone other than your prescribing physician to get sufficient pain relief from medications? (i.e., another doctor, the Emergency Room, friends, street sources)	1☐	2☐	3☐	4☐	5☐

**D. C2TKNMEDURXWY**<br>In the past 30 days, how often have you taken your medications differently from how they are prescribed?	1☐	2☐	3☐	4☐	5☐
**E. C2THGTHURT**<br>In the past 30 days, how often have you seriously thought about hurting yourself?	1☐	2☐	3☐	4☐	5☐
**F. C2TMTHKOPI**<br>In the past 30 days, how much of your time was spent thinking about opioid medications (having enough, taking them, dosing schedule, etc.)?	1☐	2☐	3☐	4☐	5☐
**G. C2ARGUE**<br>In the past 30 days, how often have you been in an argument?	1☐	2☐	3☐	4☐	5☐
**H. C2TRBCNTLANGER**<br>In the past 30 days, how often have you had trouble controlling your anger (e.g., road rage, screaming, etc.)?	1☐	2☐	3☐	4☐	5☐
**I. C2TKMEDSMO**<br>In the past 30 days, how often have you needed to take pain medications belonging to someone else?	1☐	2☐	3☐	4☐	5☐
**J. C2WRRHDLMED**<br>In the past 30 days, how often have you been worried about how you're handling your medications?	1☐	2☐	3☐	4☐	5☐
**K. C2OTHWRRHDLMED**<br>In the past 30 days, how often have others been worried about how you're handling your medications?	1☐	2☐	3☐	4☐	5☐
**L. C2ERCALL**<br>In the past 30 days, how often have you had to make an emergency phone call or show up at the clinic without an appointment?	1☐	2☐	3☐	4☐	5☐
**M. C2ANGRYOTH**<br>In the past 30 days, how often have you gotten angry with people?	1☐	2☐	3☐	4☐	5☐
**N. C2MOREMED**<br>In the past 30 days, how often have you had to take more of your medication than prescribed?	1☐	2☐	3☐	4☐	5☐
**O. C2BRRWPMED**<br>In the past 30 days, how often have you borrowed pain medication from someone else?	1☐	2☐	3☐	4☐	5☐
**P. C2PMEDOTHRSN**<br>In the past 30 days, how often have you used your pain medicine for symptoms other than for pain (e.g., to help you sleep, improve your mood, or relieve stress)?	1☐	2☐	3☐	4☐	5☐
**Q. C2VSTER**<br>In the past 30 days, how often have you had to visit the Emergency Room?	1☐	2☐	3☐	4☐	5☐

CHRONIC PAIN COPING INVENTORY - 42

The next series of questions ask you about non-medication self-management strategies for reducing chronic pain. *Self- management includes regular relaxation, physical activity, talking with others, and/or making time for social activities.*

58. During ***the past week***, how many days did you use each of the following at least once in the day to cope with your pain? (Note: You may have used some of these coping strategies on days that you did not have pain or to prevent or minimize pain in the future. Please indicate the number of days you used each strategy for pain, whether or not you were experiencing pain at the time).

Question	Number of Days
**A. P2RELAX**<br>Imagined a calming or distracting image to help me relax	☐0 ☐1 ☐2 ☐3 ☐4 ☐5 ☐6 ☐7
**B. P2IGNPAIN**<br>Ignored the pain	☐0 ☐1 ☐2 ☐3 ☐4 ☐5 ☐6 ☐7
**C. P2REST**<br>I took a rest	☐0 ☐1 ☐2 ☐3 ☐4 ☐5 ☐6 ☐7
**D. P2SUPTFRD**<br>I got support from a friend	☐0 ☐1 ☐2 ☐3 ☐4 ☐5 ☐6 ☐7
**E. P2ASKOTHHLP**<br>Asked someone to do something for me	☐0 ☐1 ☐2 ☐3 ☐4 ☐5 ☐6 ☐7
**F. P2WORSE**<br>Reminded myself that things could be worse	☐0 ☐1 ☐2 ☐3 ☐4 ☐5 ☐6 ☐7
**G. P2AVOID**<br>Avoided using part of my body (e.g. hand, arm, leg)	☐0 ☐1 ☐2 ☐3 ☐4 ☐5 ☐6 ☐7
**H. P2RELXMSL**<br>Focused on relaxing my muscles	☐0 ☐1 ☐2 ☐3 ☐4 ☐5 ☐6 ☐7
**I. P2STRTCH**<br>Sat on the floor ,stretched ,and held the stretch at least 10 seconds	☐0 ☐1 ☐2 ☐3 ☐4 ☐5 ☐6 ☐7
**J. P2BETTER**<br>Told myself things would get better	☐0 ☐1 ☐2 ☐3 ☐4 ☐5 ☐6 ☐7
**K. P2SPTFAM**<br>I got support from a family member	☐0 ☐1 ☐2 ☐3 ☐4 ☐5 ☐6 ☐7
**L. P2RESTMUCH**<br>I rested as much as I could	☐0 ☐1 ☐2 ☐3 ☐4 ☐5 ☐6 ☐7
**M. P2TALK**<br>I talked to someone close to me	☐0 ☐1 ☐2 ☐3 ☐4 ☐5 ☐6 ☐7
**N. P2CALLFRD**<br>Called a friend on the phone to help me feel better	☐0 ☐1 ☐2 ☐3 ☐4 ☐5 ☐6 ☐7
**O. P2THTGOOD**<br>Thought about all the good things I have	☐0 ☐1 ☐2 ☐3 ☐4 ☐5 ☐6 ☐7
**P. P2HLPTASK**<br>Asked for help with a chore or task	☐0 ☐1 ☐2 ☐3 ☐4 ☐5 ☐6 ☐7
**Q. P2PAINBET**<br>Told myself my pain would get better	☐0 ☐1 ☐2 ☐3 ☐4 ☐5 ☐6 ☐7
**R. P2NOINTFACT**<br>I didn't let the pain interfere with my activities	☐0 ☐1 ☐2 ☐3 ☐4 ☐5 ☐6 ☐7
**S. P2AERBEXE**<br>Engaged in aerobic exercise (exercise that made my heart beat faster) for at least 15 min.	☐0 ☐1 ☐2 ☐3 ☐4 ☐5 ☐6 ☐7
**T. P2LMTWLK**<br>Limited my walking because of pain	☐0 ☐1 ☐2 ☐3 ☐4 ☐5 ☐6 ☐7
**U. P2NPAPAIN**<br>Just didn't' pay attention to the pain	☐0 ☐1 ☐2 ☐3 ☐4 ☐5 ☐6 ☐7
**V. P2WLKLMP**<br>Walked with a limp to decrease the pain	☐0 ☐1 ☐2 ☐3 ☐4 ☐5 ☐6 ☐7

**W. P2MEDRELX**<br>Meditated to relax	☐0 ☐1 ☐2 ☐3 ☐4 ☐5 ☐6 ☐7
**X. P2LAYBACK**<br>Lay on my back, stretched, and held the stretch at least 10 seconds	☐0 ☐1 ☐2 ☐3 ☐4 ☐5 ☐6 ☐7
**Y. P2HLDPST**<br>Held part of my body (e.g. arm) in a special position	☐0 ☐1 ☐2 ☐3 ☐4 ☐5 ☐6 ☐7
**Z. P2HLPLIFT**<br>Asked for help in carrying, lifting or pushing something	☐0 ☐1 ☐2 ☐3 ☐4 ☐5 ☐6 ☐7
**AA. P2EXEIMPV**<br>Exercised to improve my overall physical condition for at least 5 minutes	☐0 ☐1 ☐2 ☐3 ☐4 ☐5 ☐6 ☐7
**BB. P2TLKSUPT**<br>Talked to a friend or family member for support	☐0 ☐1 ☐2 ☐3 ☐4 ☐5 ☐6 ☐7
**CC. P2RMDOTHWRS**<br>Reminded myself that there are people who are worse off than I am	☐0 ☐1 ☐2 ☐3 ☐4 ☐5 ☐6 ☐7
**DD. P2LMTSTNDTM**<br>Limited my standing time	☐0 ☐1 ☐2 ☐3 ☐4 ☐5 ☐6 ☐7
**EE. P2LAYDWN**<br>Lay down on a bed	☐0 ☐1 ☐2 ☐3 ☐4 ☐5 ☐6 ☐7
**FF. P2AVDPHYACT**<br>Avoided some physical activities (lifting, pushing, carrying)	☐0 ☐1 ☐2 ☐3 ☐4 ☐5 ☐6 ☐7
**GG. P2SHRELAX**<br>Used self-hypnosis to relax	☐0 ☐1 ☐2 ☐3 ☐4 ☐5 ☐6 ☐7
**HH. P2KPTGO**<br>I just kept going	☐0 ☐1 ☐2 ☐3 ☐4 ☐5 ☐6 ☐7
**II. P2STRCHMSCL**<br>Stretched the muscles where I hurt and held the stretch for at least 10 seconds	☐0 ☐1 ☐2 ☐3 ☐4 ☐5 ☐6 ☐7
**JJ. P2AVDACT**<br>Avoided activity	☐0 ☐1 ☐2 ☐3 ☐4 ☐5 ☐6 ☐7
**KK. P2RMREST**<br>Went into a room by myself to rest	☐0 ☐1 ☐2 ☐3 ☐4 ☐5 ☐6 ☐7
**LL. P2DPBRTHRLX**<br>Used deep, slow breathing to relax	☐0 ☐1 ☐2 ☐3 ☐4 ☐5 ☐6 ☐7
**MM. P2EXEMSCLBK**<br>Exercised to strengthen the muscles in my back for at least 1 minute	☐0 ☐1 ☐2 ☐3 ☐4 ☐5 ☐6 ☐7
**NN. P2ASKGETSMTH**<br>Asked someone to get me something (e.g. medicine, food, drink)	☐0 ☐1 ☐2 ☐3 ☐4 ☐5 ☐6 ☐7
**OO. P2PAINOAFFCT**<br>Did not let the pain affect what I was doing	☐0 ☐1 ☐2 ☐3 ☐4 ☐5 ☐6 ☐7
**PP. P2LAYDWSOFA**<br>Lay down on a sofa	☐0 ☐1 ☐2 ☐3 ☐4 ☐5 ☐6 ☐7

FACIT-Sp-Ex (version 4)

59. Now I am going to ask you a few questions about your spirituality. Please indicate how much you agree with each statement by marking one box per row.

Statement	Not at all	A little bit	Somewhat	Quite a bit	Very much
**A. F2STRGFAITH**<br>I find strength in my faith or spiritual beliefs	1☐	2☐	3☐	4☐	5☐
**B. F2HIGHPW**<br>I feel connected to a higher power (or God)	1☐	2☐	3☐	4☐	5☐

PAIN SELF EFFICACY QUESTIONNAIRE (PSEQ)

60. Please rate how confident you are that you can do the following things at present, despite the pain. To indicate your answer circle one of the numbers on the scale under each item, where 0 = not at all confident and 6 = completely confident. Remember, we are not asking whether or not you have been doing these things, but rather how confident you are that you can do them at present, despite the pain.

**Question**	**Not at all Confident**						**Completely Confident**
**A. P2ENJOY**<br>I can enjoy things, despite the pain.	0☐	1☐	2☐	3☐	4☐	5☐	6☐
**B. P2DOCHORE**<br>I can do most of the household chores (e.g. tidying-up, washing dishes, etc.), despite the pain.	0☐	1☐	2☐	3☐	4☐	5☐	6☐
**C. P2SOCFRND**<br>I can socialize with my friends or family members as often as I used to do, despite the pain.	0☐	1☐	2☐	3☐	4☐	5☐	6☐
**D. P2COPEPAIN**<br>I can cope with my pain in most situations.	0☐	1☐	2☐	3☐	4☐	5☐	6☐
**E. P2DOWORK**<br>I can do some form of work, despite the pain. ("work" includes housework, paid and unpaid work).	0☐	1☐	2☐	3☐	4☐	5☐	6☐
**F. P2DOMTHG**<br>I can still do many of the things I enjoy doing, such as hobbies or leisure activity, despite the pain.	0☐	1☐	2☐	3☐	4☐	5☐	6☐
**G. P2COPEWOMED**<br>I can cope with my pain without medication.	0☐	1☐	2☐	3☐	4☐	5☐	6☐
**H. P2ACCMPGOAL**<br>I can still accomplish most of my goals in life, despite the pain.	0☐	1☐	2☐	3☐	4☐	5☐	6☐
**I. P2LIVENMLF**<br>I can live a normal lifestyle, despite the pain.	0☐	1☐	2☐	3☐	4☐	5☐	6☐
**J. P2MOREACTV**<br>I can gradually become more active, despite the pain**.**	0☐	1☐	2☐	3☐	4☐	5☐	6☐

PATIENT-REPORTED OUTCOME MEASUREMENT INFORMATION SYSTEM 29-ITEM HEALTH PROFILE (PROMIS-29) AND SEXUAL FUNCTIONING

61. The next sets of questions ask you about your activity level, emotional well-being, and pain level. Please respond to each question or statement by marking one box per row.

**Physical Function**	**Without any difficulty**	**With a little difficulty**	**With some difficulty**	**With much difficulty**	**Unable to do**
**A. P2CHORE**<br>Are you able to do chores such as vacuuming or yard work?	1☐	2☐	3☐	4☐	5☐
**B. P2STAIRS**<br>Are you able to go up and down stairs at a normal pace?	1☐	2☐	3☐	4☐	5☐
**C. P2WALK**<br>Are you able to go for a walk of at least 15 minutes?	1☐	2☐	3☐	4☐	5☐

**D. P2ERRANDS**<br>Are you able to run errands and shop?	1☐	2☐	3☐	4☐	5☐
**<u>Anxiety</u>**	**Never**	**Rarely**	**Sometimes**	**Often**	**Always**
In the past 7 ***days***….					
**E. P2FEAR**<br>I felt fearful…..	1☐	2☐	3☐	4☐	5☐
**F. P2FOCUS**<br>I found it hard to focus on anything other than my anxiety…	1☐	2☐	3☐	4☐	5☐
**G. P2WORRY**<br>My worries overwhelmed me……	1☐	2☐	3☐	4☐	5☐
**H. P2UNEASY**<br>I felt uneasy……	1☐	2☐	3☐	4☐	5☐
**<u>Depression</u>**	**Never**	**Rarely**	**Sometimes**	**Often**	**Always**
In the past 7 ***days***….					
**I. P2WRTHLS**<br>I felt worthless……	1☐	2☐	3☐	4☐	5☐
**J. P2HLPLS**<br>I felt helpless…….	1☐	2☐	3☐	4☐	5☐
**K. P2DEPRESS**<br>I felt depressed……	1☐	2☐	3☐	4☐	5☐
**L. P2HOPELS**<br>I felt hopeless…….	1☐	2☐	3☐	4☐	5☐
**<u>Fatigue</u>**	**Not at all**	**A little bit**	**Somewhat**	**Quite a bit**	**Very much**
During the past 7 ***days***….					
**M. P2FLTFTG**<br>I felt fatigued……	1☐	2☐	3☐	4☐	5☐
**N. P2TRBTHK**<br>I have trouble <u>starting</u> thinking because I am tired……	1☐	2☐	3☐	4☐	5☐
In the past 7 ***days***….					
**O. P2RUNDWN**<br>How run down did you feel on average?	1☐	2☐	3☐	4☐	5☐
**P. P2HOWFTG**<br>How fatigued were you on average?	1☐	2☐	3☐	4☐	5☐
**<u>Sleep Disturbance</u>**	**Very poor**	**Poor**	**Fair**	**Good**	**Very good**
In the past 7 ***days***….					
**Q. P2SLPQLT**<br>My sleep quality was…..	1☐	2☐	3☐	4☐	5☐
In the past 7 ***days***….	**Not at all**	**A little bit**	**Somewhat**	**Quite a bit**	**Very much**
**R. P2SLPRFRSH**<br>My sleep was refreshing…..	1☐	2☐	3☐	4☐	5☐
**S. P2PRBSLP**<br>I had a problem with my sleep…..	1☐	2☐	3☐	4☐	5☐
**T. P2DFTSLP**<br>I had difficulty falling asleep….	1☐	2☐	3☐	4☐	5☐
**<u>Satisfaction with Social Role</u>**	**Not at all**	**A little bit**	**Somewhat**	**Quite a bit**	**Very much**
In the past 7 ***days***….					
**U. P2SATWRK**<br>I am satisfied with how much work I can do (include work at home)	1☐	2☐	3☐	4☐	5☐
**V. P2SATWKABLTY**<br>I am satisfied with my ability to work (include work at home)	1☐	2☐	3☐	4☐	5☐

**W. P2SATRESPNS**<br>I am satisfied with my ability to do regular personal and household responsibilities	1☐	2☐	3☐	4☐	5☐
**X. P2SATPERFM**<br>I am satisfied with my ability to perform my daily routines	1☐	2☐	3☐	4☐	5☐
**Pain Interference**	**Not at all**	**A little bit**	**Somewhat**	**Quite a bit**	**Very much**
In the past *7 days*….					
**Y. P2PAINACT**<br>How much did pain interfere with your day to day activities?	1☐	2☐	3☐	4☐	5☐
**Z. P2PAINWRK**<br>How much did pain interfere with work around the home?	1☐	2☐	3☐	4☐	5☐
**AA. P2PAINSOC**<br>How much did pain interfere with your ability to participate in social activities?	1☐	2☐	3☐	4☐	5☐
**BB. P2PAINCHORE**<br>How much did pain interfere with your household chores?	1☐	2☐	3☐	4☐	5☐
**Pain Intensity**					
In the past *7 days*….					
**CC. P2PAINRT**<br>How would you rate your pain on average on a scale of 1 to 10?<br>**1 2 3 4 5 6 7 8 9 10**<br>|-----------|-----------|-----------|-----------|-----------|-----------|-----------|-----------|-----------| **No Pain** … **Worst Imaginable Pain**<br>Answer: ______					

PROMIS

Next are just a few questions about your sex life.

62. P2SEXACT
In the past ***30 days…*** Have you had any type of sexual activity with another person (including your partner)?

1☐ Yes
2☐ No

63. P2STSSEXLF
In the past ***30 days…*** How satisfied have you been with your sex life?

1☐ Not at all
2☐ A little bit
3☐ Somewhat
4☐ Quite a bit
5☐ Very

SOCIAL SUPPORT

64. S2PSNHELP
How many people will help you with practical things when you need it, like giving you rides, helping with baby-sitting, a quick loan, and so forth?

______ number of people

65. S2FAMCNTCT
About how many family members (including spouse) and friends, lovers or partners would you say you regularly have contact with? (By regularly, we mean you see them or talk on the phone with them at least twice a month on average.)

______ number of people

66. S2PSNTALK
How many people do you have in your life to talk to when you worry about personal problems, such as family or work?

______ number of people

PATIENT HEALTH QUESTIONNAIRE-9 (PHQ-9)

67. Now I am going to ask you about your mood in the past two weeks.
Over the last 2 weeks, how often have you been bothered by any of the following problems?

	Not at all	Several days	More than half the days	Nearly every day
A. **P2LILPLS**<br>Little interest or pleasure in doing things	0☐	1☐	2☐	3☐
B. **P2DEPRES**<br>Feeling down, depressed, or hopeless	0☐	1☐	2☐	3☐
C. **P2TRBSLP**<br>Trouble falling or staying asleep, or sleeping too much	0☐	1☐	2☐	3☐
D. **P2TIRED**<br>Feeling tired or having little energy	0☐	1☐	2☐	3☐
E. **P2APPET**<br>Poor appetite or overeating	0☐	1☐	2☐	3☐
F. **P2FELBAD**<br>Feeling bad about yourself — or that you are a failure or have let yourself or your family down	0☐	1☐	2☐	3☐
G. **P2CONCEN**<br>Trouble concentrating on things, such as reading the newspaper or watching television	0☐	1☐	2☐	3☐

H. **P2SLOW**<br>Moving or speaking so slowly that other people could have noticed? Or the opposite — being so fidgety or restless that you have been moving around a lot more than usual	0☐	1☐	2☐	3☐
I. **P2HURT**<br>Thoughts that you would be better off dead or of hurting yourself in some way	0☐	1☐	2☐	3☐

J. **P2DIFWOR**<br>**IF P2LILPLS THRU P2HURT=0 THEN SKIP:**<br>If you checked off <u>any</u> problems, how <u>difficult</u> have these problems made it for you to do your work, take care of things at home, or get along with other people?	Not difficult at all<br>1☐	Somewhat difficult<br>2☐	Very difficult<br>3☐	Extremely difficult<br>4☐

PROMIS GLOBAL HEALTH (QoL)

The following are questions about your general quality of life.

68. Please respond to each item by marking one box per row.

Statement	Poor	Fair	Good	Very good	Excellent
A. **Q2HELTH**<br>In general, would you say your health is:	1☐	2☐	3☐	4☐	5☐
B. **Q2QLTLIFE**<br>In general, would you say your quality of life is:	1☐	2☐	3☐	4☐	5☐
C. **Q2RTPHLTH**<br>In general, how would you rate your physical health?	1☐	2☐	3☐	4☐	5☐
D. **Q2RTMHLTH**<br>In general, how would you rate your mental health, including your mood and your ability to think?	1☐	2☐	3☐	4☐	5☐
E. **Q2RTSTSSOC**<br>In general, how would you rate your satisfaction with your social activities and relationships?	1☐	2☐	3☐	4☐	5☐
F. **Q2RTSOCACT**<br>In general, please rate how well you carry out your usual social activities and roles. (This includes activities at home, at work and in your	1☐	2☐	3☐	4☐	5☐

community, and responsibilities as a parent, child, spouse, employee, friend, etc.).					
	**Not at all**	**A little**	**Moderately**	**Mostly**	**Completely**
G. **Q2PHYACT**<br>To what extent are you able to carry out your everyday physical activities such as walking, climbing stairs, carrying groceries, or moving a chair?	1☐	2☐	3☐	4☐	5☐
**IN THE PAST 7 DAYS….**	**Always**	**Often**	**Sometimes**	**Rarely**	**Never**
H. **Q2EMPRB**<br>How often have you been bothered by emotional problems such as feeling anxious, depressed or irritable?	1☐	2☐	3☐	4☐	5☐
I. **Q2RTFTG**<br>How would you rate your fatigue on average?	**Very severe**	**Severe**	**Moderate**	**Mild**	**None**
	1☐	2☐	3☐	4☐	5☐

SUBSTANCE USE QUESTION NIDA-MODIFIED ASSIST

Next we are going to ask you some questions about substance use.
As a reminder, your answers are completely confidential.

69. In the past 6 months, which of the following substances have you used?

Question	Yes	No
**A. A2EVEMAR**<br>**Cannabis** (marijuana, pot, grass, hash, etc.)	1☐	2☐
**B. A2EVECOC**<br>**Cocaine** (coke, crack, etc.)	1☐	2☐
**C. A2EVERXSTI**<br>**Prescription stimulants** (Ritalin, Concerta, Dexedrine, Adderall, diet pills, etc.)	1☐	2☐
**D. A2EVEMET**<br>**Methamphetamine** (speed, crystal meth, ice, etc.)	1☐	2☐
**E. A2EVEINH**<br>**Inhalants** (nitrous oxide, glue, gas, paint thinner, etc.)	1☐	2☐
**F. A2EVESED**<br>**Sedatives or sleeping pills** (Valium, Serepax, Ativan, Xanax, Librium, Rohypnol, GHB, etc.)	1☐	2☐
**G. A2EVEHAL**<br>**Hallucinogens** (LSD, acid, mushrooms, PCP, Special K, ecstasy, etc.)	1☐	2☐
**H. A2EVEHER**<br>**Street opioids** (heroin, opium, etc.)	1☐	2☐
**I. A2EVERXOPI**<br>**Prescription opioids** (fentanyl, oxycodone [OxyContin, Percocet], hydrocodone [Vicodin], methadone, buprenorphine, etc.)	1☐	2☐

**J. A2EVEOTH**<br>**Other – Specify:**	1☐	2☐

IF **A2EVEMAR THRU A2EVEOTH = 1 THEN GO TO A2USEDRG3M**
IF **A2EVEMAR THRU A2EVEOTH = 2 THEN SKIP TO S2SMK100**

70. Ask the following questions for each drug mentioned above:

Question	Never	Once or Twice	Monthly	Weekly	Daily or Almost Daily
**A. A2USEDRG3M**<br>*In the past 3 months*, how often have you used (insert name of drug)?<br>IF **A2USEDRG3M = 1 THEN SKIP TO A2FRDCRNDRG**	1☐	2☐	3☐	4☐	5☐
**B. A2DESRUSEDRG**<br>In the past 3 months, how often have you had a strong desire or urge to use (insert name of drug)?	1☐	2☐	3☐	4☐	5☐
**C. A2OFTUSEDRG**<br>In the past 3 months, how often has your use of (insert name of drug) led to health, social, legal or financial problems?	1☐	2☐	3☐	4☐	5☐
**D. A2FAILDO**<br>In the past 3 months, how often have you failed to do what was normally expected of you because of your use of (insert name of drug)?	1☐	2☐	3☐	4☐	5☐

Ask Questions “**E**”& “**F**” for all substances *used in the last 6 months*:	**NO**	**YES**, but not in the last 3 months	**YES**, in the past three months
**E. A2FRDCRNDRG**<br>Has a friend or relative or anyone else ever expressed concern about your use of (insert name of drug)?	1☐	2☐	3☐
**F. A2FAILCUTDRG**<br>Have you ever tried and failed to control, cut down, or stop using (insert name of drug)?	1☐	2☐	3☐

IF A2EVEMAR =1, AND A2USEDRG3M_MAR = 2,3,4,5, THEN A2MAR30D …

(IF A2EVEMAR = 2 OR A2USEDRG3M_MAR = 1 THEN SKIP TO S2SMK100)

71. **A2MAR30D**
Did you use marijuana in past 30 days for medical purposes, as prescribed by a health care provider?

1☐ Yes
2☐ No
3☐ Sometimes for medical reasons, sometimes not
97 ☐ Don't Know/Refused

71A. **A2MARRSN**
Please indicate all the health or medical reasons for which you used marijuana in the past 30 days.

1☐ Pain
2☐ Appetite
3☐ Anxiety
4☐ Depression
5☐ Sleep
7☐ Other (Specify): ___________________
97 ☐ Don't Know/Refused

CIGARETTES/SMOKING

Now we are going to ask you a few questions about smoking.

72. **S2SMK100**
Have you smoked at least 100 cigarettes (5 packs or more) in your lifetime?
1☐ Yes
2☐ No →**SKIP TO S2ECIG30D**

73. **S2SMKAVGDY**
On average, how many cigarettes per day do you usually smoke (or did you usually smoke when you did smoke)?
___ ___ # of Cigarettes

74. **S2DYSMK30D**
In the past 30 days, on how many days did you smoke at least one cigarette?
___ ___# Days ENTER "0" IF YOU DO NOT CURRENTLY SMOKE

75. **S2ECIG30D**
In the past 30 days, on how many days did you use an E-cigarette/E-Hookah/Vape pen?
___ ___ # of Days ENTER "0" IF DO NOT CURRENTLY USE E-CIG

ALCOHOL USE

1 drink is equal to a 12 ounce can of beer, a 5 ounce glass of wine, a 1.5 ounce shot of liquor, or an 8-9 ounce malt liquor

IF D2GENDER ≠ 1 OR (D2GENDER = 1 AND D2AGE > 65) THEN SKIP TO T2ALCF4

76. T2ALCM5

How many times in the past three months have you had 5+ drinks containing alcohol in a day?

___ ___ # of Days

IF D2GENDER = 1 AND D2AGE <= 65 THEN SKIP TO T2DRINKS

77. T2ALCF4

How many times in the past three months have you had 4+ drinks containing alcohol in a day?

___ ___ # of Days

78. T2DRINKS

On a typical drinking day, how many drinks do you have?

___ ___ # of Drinks ENTER “0” IF DO NOT DRINK

79. T2ALC1WK

On average, how many days a week do you have an alcoholic drink?

___ ___ # of Days ENTER “0” IF DO NOT DRINK

IF MEN,
AND >4 DRINKS PER DAY (**T2DRINKS** > 4), OR
>14 DRINKS PER WEEK (**T2DRINKS * T2ALC1WK**), THEN ASK THE FOLLOWING TWO QUESTIONS

80. T2HURTMALC

In the past 6 months, have you sometimes been under the influence of alcohol in situations where you could have caused an accident or gotten hurt?

1☐ Yes
2☐ No

81. T2MOREMALC

Have there often been times in the past 6 months when you had a lot more to drink than you intended to have?

1☐ Yes
2☐ No

IF MEN>=65 OR WOMEN,
AND > 3 DRINKS PER DAY (**T2DRINKS** > 3), OR
> 7 DRINKS PER WEEK (**T2DRINKS * T2ALC1WK**), THEN ASK THE FOLLOWING TWO QUESTIONS

82. T2HURTFALC

In the past 6 months, have you sometimes been under the influence of alcohol in situations where you could have caused an accident or gotten hurt?

1☐ Yes
2☐ No

83. T2MOREFALC

Have there often been times in the past 6 months when you had a lot more to drink than you intended to have?

1☐ Yes
2☐ No

84. **H2PCPTLK**

Have you talked to your Primary Care Provider about drug or alcohol problems in the past 6 months?

1☐ Yes
2☐ No **→SKIP TO C2GREET**
97☐ Don't Know/Refused

85. **H2SPKDOC**

How often in the past 6 months have you spoken with your Primary Care Provider about this?

1☐ Once
2☐ More than once

PATIENT PROVIDER COMMUNICATION

Communication Assessment Tool

We would like to know how you feel about the way your primary care physician has communicated with you in your last couple of visits. Your answers are completely confidential, so please be as open and honest as you can.

86. Please rate your physician's communication with you by marking **one box per row.**

Statement	Poor	Fair	Good	Very good	Excellent
**My doctor**					
**A. C2GREET** Greeted me in a way that made me feel comfortable	1☐	2☐	3☐	4☐	5☐
**B. C2TXRSPT** Treated me with respect	1☐	2☐	3☐	4☐	5☐
**C. C2INSTIDEA** Showed interest in my ideas about my health	1☐	2☐	3☐	4☐	5☐
**D. C2UNDHLTCNR** Understood my main health concerns	1☐	2☐	3☐	4☐	5☐
**E. C2ATTEN** Paid attention to me (looked at me, listened carefully)	1☐	2☐	3☐	4☐	5☐
**F. C2NOINTRPT** Let me talk without interruptions	1☐	2☐	3☐	4☐	5☐
**G. C2GVINFO** Gave me as much information as I wanted	1☐	2☐	3☐	4☐	5☐
**H. C2TLKUNDSTD** Talked in terms I could understand	1☐	2☐	3☐	4☐	5☐
**I. C2CHKUNDSTD** Checked to be sure I understood everything	1☐	2☐	3☐	4☐	5☐
**J. C2ENCGASKQ** Encouraged me to ask questions	1☐	2☐	3☐	4☐	5☐
**K. C2INVDEC** Involved me in decisions as much as I wanted	1☐	2☐	3☐	4☐	5☐
**L. C2DISFU** Discussed next steps, including any follow-up plans	1☐	2☐	3☐	4☐	5☐
**M. C2CARECNR** Showed care and concern	1☐	2☐	3☐	4☐	5☐
**N. C2SPTTIME** Spent the right amount of time with me	1☐	2☐	3☐	4☐	5☐

PEPPI Perceived Efficacy in Patient-Physician Interactions Questionnaire

87. Next, please rate your confidence level in your communication with your physician on a scale of 1 to 5, with 1 being "not at all confident" and 5 being "very confident".

.

**Statement**	**Not at all confident**				**Very Confident**
**How confident are you in your ability to:**					
**A. P2DRATTN**<br>Get a doctor to pay attention to what you have to say?	1☐	2☐	3☐	4☐	5☐
**B. P2KNWASKDC**<br>Know what questions to ask a doctor?	1☐	2☐	3☐	4☐	5☐
**C. P2DRANSQST**<br>Get a doctor to answer all of your questions?	1☐	2☐	3☐	4☐	5☐
**D. P2ASKDRHLTH**<br>Ask a doctor questions about your chief health concern?	1☐	2☐	3☐	4☐	5☐
**E. P2VSMSTTDR**<br>Make the most of your visit with the doctor?	1☐	2☐	3☐	4☐	5☐
**F. P2DRSERHLTH**<br>Get a doctor to take your chief health concerns seriously?	1☐	2☐	3☐	4☐	5☐
**G. P2UNDSTDR**<br>Understand what a doctor tells you?	1☐	2☐	3☐	4☐	5☐
**H. P2GTDRDOSM**<br>Get a doctor to do something about your chief health concern?	1☐	2☐	3☐	4☐	5☐
**I. P2EXPDRHLTHCRN**<br>Explain your chief health concern to a doctor?	1☐	2☐	3☐	4☐	5☐
**J. P2ASKMINF**<br>Ask a doctor for more information if you don’t understand what he or she said?	1☐	2☐	3☐	4☐	5☐

You have completed the 12 month questionnaire.

Thank you!

List of common prescription opiate medications

Buprenorphine
Buprenorphine/Naloxone
Butalbital with Codeine
Butrans
Codeine
Darvocet
Darvon
Dihydrocodeine
Duragesic **(**Fentanyl transdermal)
Fentanyl
Fiorinal with codeine
Hydrocodone
Kadian
Levorphanol
Meperidine or Demerol
Methadone
Morphine
MS Contin
Norco
Nubain
Opana
Oramorph
Oxycodone
Oxycontin
OxyFast OxyIR
Oxymorphone
Pentazocine
Percocet
Propoxyphene
Reprexain
Roxanol
Roxicodone
Suboxone
Talwin
Tramadol or Ultram
Tylenol #3
Tylox
Vicodin
Vicoprofen
Zubsolv
